# A Compartment Model of VEGF Distribution in Humans in the Presence of Soluble VEGF Receptor-1 Acting as a Ligand Trap

**DOI:** 10.1371/journal.pone.0005108

**Published:** 2009-04-08

**Authors:** Florence T. H. Wu, Marianne O. Stefanini, Feilim Mac Gabhann, Aleksander S. Popel

**Affiliations:** 1 Department of Biomedical Engineering, Johns Hopkins University School of Medicine, Baltimore, Maryland, United States of America; 2 Department of Biomedical Engineering and Robert M. Berne Cardiovascular Research Center, University of Virginia, Charlottesville, Virginia, United States of America; Universidad Europea de Madrid, Spain

## Abstract

Vascular endothelial growth factor (VEGF), through its activation of cell surface receptor tyrosine kinases including VEGFR1 and VEGFR2, is a vital regulator of stimulatory and inhibitory processes that keep angiogenesis – new capillary growth from existing microvasculature – at a dynamic balance in normal physiology. Soluble VEGF receptor-1 (sVEGFR1) – a naturally-occurring truncated version of VEGFR1 lacking the transmembrane and intracellular signaling domains – has been postulated to exert inhibitory effects on angiogenic signaling via two mechanisms: direct sequestration of angiogenic ligands such as VEGF; or dominant-negative heterodimerization with surface VEGFRs. In pre-clinical studies, sVEGFR1 gene and protein therapy have demonstrated efficacy in inhibiting tumor angiogenesis; while in clinical studies, sVEGFR1 has shown utility as a diagnostic or prognostic marker in a widening array of angiogenesis–dependent diseases. Here we developed a novel computational multi-tissue model for recapitulating the dynamic systemic distributions of VEGF and sVEGFR1. Model features included: physiologically-based multi-scale compartmentalization of the human body; inter-compartmental macromolecular biotransport processes (vascular permeability, lymphatic drainage); and molecularly-detailed binding interactions between the ligand isoforms VEGF_121_ and VEGF_165_, signaling receptors VEGFR1 and VEGFR2, non-signaling co-receptor neuropilin-1 (NRP1), as well as sVEGFR1. The model was parameterized to represent a healthy human subject, whereupon we investigated the effects of sVEGFR1 on the distribution and activation of VEGF ligands and receptors. We assessed the healthy baseline stability of circulating VEGF and sVEGFR1 levels in plasma, as well as their reliability in indicating tissue-level angiogenic signaling potential. Unexpectedly, simulated results showed that sVEGFR1 – acting as a diffusible VEGF sink alone, i.e., without sVEGFR1-VEGFR heterodimerization – did not significantly lower interstitial VEGF, nor inhibit signaling potential in tissues. Additionally, the sensitivity of plasma VEGF and sVEGFR1 to physiological fluctuations in transport rates may partially account for the heterogeneity in clinical measurements of these circulating angiogenic markers, potentially hindering their diagnostic reliability for diseases.

## Introduction

### The VEGF System in Angiogenesis

Vascular endothelial growth factor (VEGF) – a family of cytokines that include VEGF-A, VEGF-B and placental growth factor, PlGF – is central to the regulation of competing stimulatory and inhibitory biochemical reactions that keep vascular angiogenesis – the growth of new blood capillaries from existing microvasculature – at a critical homeostasis in normal physiology [1]–[3]. VEGF_121_, VEGF_165_, VEGF_189_ are considered the predominant pro-angiogenic isoforms of human VEGF-A (UniProt accession P15692); their mRNA expression levels in muscle tissues have been found to range from 3∶4∶3 in humans [4] to 8∶77∶15 (VEGF_120_∶VEGF_164_∶VEGF_188_) in mice [5]. Their endogenous prevalence makes VEGF_121_ and VEGF_165_ common therapeutic agents in pro-angiogenic treatment for ischemic pathologies such as peripheral arterial disease (PAD) and coronary artery disease (CAD) [1], [6]. Once secreted into the extracellular matrix, VEGF_121_ is generally considered a freely diffusible isoform, while VEGF_165_ and VEGF_189_ can be sequestered through its heparin-binding domain at interstitial and cell surface proteoglycans in significant quantities. Among the major endothelial cell surface receptor targets for these VEGF isoforms are: the tyrosine kinases VEGFR1 (Flt-1; UniProt accession P17948-1) and VEGFR2 (mouse Flk-1; human KDR; UniProt accession P35968); as well as the co-receptor neuropilin-1 (NRP1; UniProt accession O14786), which couples directly with VEGFR1, and indirectly with VEGFR2 through non-overlapping binding sites on VEGF_165_ [7].

### Soluble VEGF Receptor-1

Soluble VEGFR1 (sVEGFR1; UniProt accession P17948-2), also known as soluble fms-like tyrosine kinase receptor-1 (sFlt-1), is a naturally-occurring truncated 110-kDa version of the 180-kDa membrane-spanning VEGFR1 [8], [9], derived predominantly from alternative splicing of the VEGFR1 gene, but possibly with contributions from proteolytic shedding of the extracellular fragment of VEGFR1 as well [10]–[12]. While lacking the transmembrane and intracellular signaling domains of VEGFR1, sVEGFR1 retains the protein structure and molecular functionality of the first six immunoglobulin-like extracellular domains of VEGFR1: i.e., an affinity for VEGF ligands, the NRP1 co-receptor, and extracellular heparan sulfate proteoglycans, as well as a capacity for homo/hetero-dimerization [13]–[15].

Studies have shown that the anti-angiogenic property of sVEGFR1 plays a critical role in the normal physiology of maintaining corneal avascularity [16] and in the pathology of the pregnancy disorder, pre-eclampsia [17], [18]. Recently, sVEGFR1 has been implicated in the impaired ischemic muscle angiogenesis of PAD. In diabetic mouse models of PAD, muscle expression of VEGF and sVEGFR1 both increased after surgically-induced hindlimb ischemia [19]. In clinical trials of therapeutic angiogenesis for CAD and PAD, the inconsistent efficacy of VEGF protein/gene therapy prompted a hypothesis that patients of these atherosclerotic vascular diseases may suffer from ligand insensitivity due to impaired receptor signaling or altered expression of antagonists (e.g., sVEGFR1), rather than simply a lack of angiogenic growth factors themselves [6].

It has been postulated that sVEGFR1 inhibits VEGF-signaling via two molecular mechanisms – (1) direct ligand trapping of VEGF ligands; and (2) heterodimerization with surface VEGFR monomers to form dominant-negative receptor dimers [9], [20] – though their relative functional contributions *in vivo* are not known. Nonetheless, pre-clinical studies on sVEGFR1 gene/protein therapy have already shown success in interfering with VEGF-dependent pathological processes including tumor angiogenesis [21]–[30], ocular neovascularization [31], [32], inflammation [33]–[35] and edema [36].

Furthermore, researchers are increasingly finding clinical utility for sVEGFR1, either independently or in combination with circulating VEGF or PlGF, as diagnostic and prognostic markers for a diversity of angiogenesis-dependent medical conditions: from astrocytic gliomas [37], primary breast cancer [38], acute myeloid leukemia [39], to sepsis [40], and pre-eclampsia [41], [42]. In PAD, while plasma VEGF has been correlated with disease severity [43], there is contradicting evidence regarding whether plasma sVEGFR1 level is significantly lowered relative to healthy controls [43]–[46]. A key issue hindering the reliability of circulating sVEGFR1 levels as surrogate markers of the angiogenic status in angiogenesis-dependent pathologies is the striking inter-study and intra-study heterogeneity in clinical measurements of plasma sVEGFR1 for healthy control subjects [39], [40], [42]–[52] – a variability of over several orders of magnitude which may be attributable to inter-study methodological differences, as well as natural variation at physiological baseline among individuals within control groups [44], [53].

Hence we identified the need for a computational model that predicts the systemic distributions and molecular interactions of VEGF and sVEGFR1 across the human body, to serve as a platform for the mechanistic study of sVEGFR1's purported anti-angiogenic properties, as well as the pre-clinical study of sVEGFR1 as a disease marker and therapeutic agent.

### Computational Systems Biology Modeling

We have previously developed several computational models of the *in vivo* biochemical interactions between VEGF_121_, VEGF_165_, VEGFR1, VEGFR2, NRP1, and the interstitial matrix in skeletal muscle, including: a spatially-averaged single-tissue model of the human vastus lateralis muscle at rest [54]; and several 3D models for predicting spatial molecular gradients and intra-muscular pro-angiogenic treatment outcomes in resting and exercising rat extensor digitorum longus muscle [55]–[58]. A recent extension has been to develop a multi-tissue lumped-compartment framework to describe the systemic distributions of VEGF across the human body; a first application to study human breast cancer introduced macromolecular transport between the blood, healthy solid tissue and tumor compartments through vascular permeability [59]. In the present study, further model extensions have been made to include: (i) sVEGFR1, an endogenous antagonist of VEGF, into the network of molecular interactions; as well as (ii) lymphatic drainage, as a second mode of macromolecular transport from the solid tissue compartments into the blood.

### Lymphatic Drainage of sVEGFR1

The lymphatic vasculature is a one-way drainage system that returns fluid and proteins from the interstitial spaces of tissues into the blood [60]. The inclusion of lymphatic drainage in the modeling of sVEGFR1 biodistribution was particularly relevant for two reasons. Firstly, relative to VEGF (∼46-kDa dimer [10]), sVEGFR1 (∼220-kDa dimer [8]) is expected to rely more on lymphatic drainage to clear its interstitial accumulation, because its larger molecular size theoretically should hinder blood capillary reabsorption (vascular permeability), and it also has relatively fewer routes of receptor-mediated internalization (especially if heterodimerization with surface receptors proves insignificant). Secondly, the lymphatic capillaries in skeletal muscle rely on the “milking effect” of rhythmical contractions and relaxations by adjacent skeletal muscle for lymph propulsion [61], [62]. As lymph flow directly transfers protein mass from the interstitial space into the blood, concentrations of VEGF and sVEGFR1 in muscle interstitia and in plasma could be sensitive to the muscle activity-dependent changes in lymph flow rates. Hence we hypothesized that the physiological activity-dependent fluctuations in lymphatic drainage rates may contribute to the clinical heterogeneity observed in plasma measurements of VEGF and sVEGFR1. The implications of such an association would be significant: (i) impaired ambulation (due to the PAD symptoms of intermittent claudication) and the associated reductions in leg muscle contractions and lymph flow rate may partially account for the tissue edema [62] and lowered plasma sVEGFR1 levels observed in PAD [44], [45]; conversely, (ii) the success of exercise rehabilitation for PAD patients can potentially be explained by the restoration of lymph flow rate with increased leg muscle contractions [61], [63], [64].

### Objectives and Achievements

In summary, the goals of this study were: (1) to develop a computational multi-tissue model for recapitulating the systemic distributions of VEGF and sVEGFR1; (2) to parameterize the model for a healthy human subject; and (3) to investigate the effects of sVEGFR1 and lymphatic drainage on the VEGF ligand-receptor system. Firstly, we explored whether sVEGFR1 – in its capacity as a VEGF sink alone (without heterodimerization with surface VEGFRs) – can demonstrate anti-angiogenic potential, e.g., lowering free VEGF availability and inhibiting signaling complex formation. Secondly, we tested the hypothesis that physiological fluctuations in transport rates – especially the muscle activity-dependent lymphatic drainage rates – contribute to the natural variability in plasma concentrations of VEGF and sVEGFR1 as observed clinically. Taken together, the results yielded an assessment of the general utility of plasma VEGF and sVEGFR1 levels as surrogate markers for the angiogenic status in diseases, specifically regarding their stability (i.e., whether their healthy benchmark levels remained steady throughout physiological perturbations in system parameters including transport rates) and reliability (i.e., whether their changes correlated with interstitial changes in angiogenic signaling potential).

## Materials and Methods

### 1. Compartmental Model Formulation

**Figure 1:**
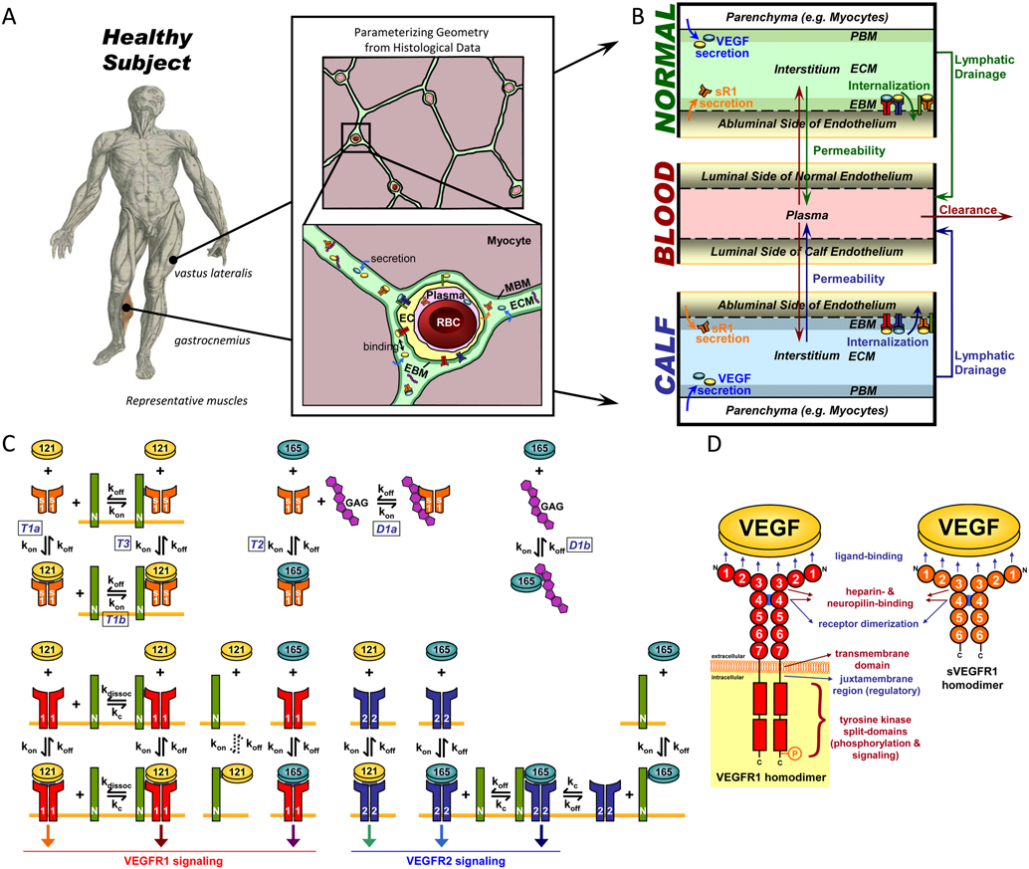
Schematic of Multi-Tissue Model of VEGF and sVEGFR1 Distributions. A. *Whole-body compartmentalization of solid tissues into ‘Calf’ vs. ‘Normal’ (rest of the body) compartments for a healthy subject*. Characteristic geometries – parenchymal cell (grayish red), interstitial space (green/blue) and capillary space (endothelial cells (EC) in yellow, plasma in pink, red blood cells (RBC) in red) volume fractions; basement membrane (BM) thicknesses; and EC surface areas – were quantified from histological cross-sections of representative human skeletal muscles (gastrocnemius and vastus lateralis). Illustrations adapted from: ‘muscle man’ series from Andreas Vesalius, De Humani Corpis Fabrica, 1543, courtesy of the National Library of Medicine; histological micrographs from Baum *et al.* J Vasc Res 2007;44:202–213. Note: BM thicknesses and molecule sizes are not drawn to scale. B. *Mass flows through 3-compartment model*. VEGF and sVEGFR1 were secreted from parenchymal and endothelial cell sources respectively. Both were internalized upon binding with abluminal endothelial surface receptors. All soluble species were subjected to lymphatic drainage from interstitial space into the blood, bidirectional permeability through the endothelia, and direct clearance from the blood. C. *Molecular Interactions between VEGF_121_ (yellow), VEGF_165_ (blue), sVEGFR1 (orange), interstitial matrix binding sites (glycosaminoglycans or “GAG”; purple), and the abluminal endothelial cell surface receptors VEGFR1 (red), VEGFR2 (blue), and NRP1 (green)*. The sVEGFR1 interactions modeled were: trapping of free VEGF_121_ (T1a) and VEGF_165_ (T2); giving NRP1s an indirect way of sequestering VEGF_121_ to the cell surface (T1b or T3); and displacing VEGF_165_ from interstitial matrix sites (D1b) through competitive binding (D1a). This model neglected sVEGFR1-heterodimerization with surface VEGFRs, thus ignoring the possible effect of sVEGFR1 in lowering the effective density of functional surface VEGFRs. Hence in this study, any effect that the presence of sVEGFR1 had on VEGF-signaling potential (i.e, the formation of VEGF-VEGFR complexes) resulted from sVEGFR1's influence on the effective concentration of interstitial free VEGF. D. *Protein domains of full-length vs. soluble human VEGFR1*. sVEGFR1's binding affinities for the VEGF ligand, interstitial matrix sites (e.g., heparan sulfate proteoglycans) and NRP1 were inferred from the identical first 6 immunoglobulin-like domains of the full-length VEGFR1.

To model the systemic distributions of VEGF and sVEGFR1 associated with healthy subjects, a physiologically-based pharmacokinetic model was constructed that divided the human body into three compartments: (1) the “*calf*”, consisting of the gastrocnemius and soleus muscles; (2) the “*normal (rest of the body)*”, consisting of all other solid tissues; and (3) the “*blood*”, that interacts with the other two compartments. The purpose of distinguishing a calf compartment was two-fold: (a) to investigate the differential effects of varying local (calf) vs. global (body) parameters in the present study; and (b) in preparation for future modelling of and comparative investigations against PAD patients whose calf muscles are common symptomatic sites of tissue ischemia and impaired angiogenesis [43], [65]). Further spatial distinctions within the tissue compartments into parenchymal space, interstitial space and capillary space were necessary to distinguish tissue-specific pools of VEGF and sVEGFR1 into free vs. matrix-bound vs. receptor-bound subpopulations. As illustrated in Fig. 1A, histological data from cross-sections of healthy human muscles were used to characterize the relevant volumes and surface areas of these spatial subdivisions for the tissue compartments.

Within the tissue compartments, free VEGF_121_ and VEGF_165_ were secreted from parenchymal cells (myocytes) in a 1∶10 ratio in correspondence with the mRNA expression ratio of freely diffusible isoforms (VEGF_120_) vs. heparin-binding isoforms (VEGF_164_+VEGF_188_) in mice [5]. Free sVEGFR1 was secreted abluminally from endothelial cells into the interstitial space (Fig. 1B); luminal secretion of sVEGFR1 was neglected in congruency with model assumptions to neglect luminal insertion of membrane-tethered VEGFRs (for which there is little quantitive evidence and will be separately investigated in further studies). Proteolytic shedding of sVEGFR1 from abluminal surface VEGFR1 was neglected in our model, as was VEGF_165_ proteolysis by plasmin and MMPs [66]. Tissue concentrations of VEGF and sVEGFR1 were then distributed – through competing binding interactions summarized in Fig. 1C – over three populations: (i) diffusing freely or as VEGF-sVEGFR1 complexes within the available interstitial fluid; (ii) sequestered in the endothelial basement membrane (EBM), extracellular matrix (ECM), and parenchymal basement membrane (PBM) regions of the interstitial space, each with distinct compositions of VEGF- and sVEGFR1-binding proteoglycans; or (iii) bound to abluminal endothelial cell surface receptors. The binding interactions of sVEGFR1, in particular, were inferred functionalities based on the first six Ig-like domains that it has in common with VEGFR1 (Fig. 1D). Since the current implementation neglected heterodimerization of sVEGFR1 with surface VEGFRs, internalization of sVEGFR1 from the endothelial surface could only occur through binding to surface NRP1s; while VEGF could be internalized through the six signaling VEGFR complexes in addition to uncoupled NRP1s (Fig. 1B,C). Additionally, the soluble volumetric species – free VEGF, free sVEGFR1 and VEGF-sVEGFR1 complex – were subjected to inter-compartmental transport processes: (i) lymphatic drainage from the interstitium into plasma; (ii) bidirectional vascular permeability between interstitial fluid and plasma; and (iii) direct clearance from plasma (Fig. 1B).

In this spatially-averaged model, gradients of soluble species were neglected within the interstitial fluid (where diffusion time was faster than reaction time as justified by Damkohler number <1 [54]) and plasma. Spatial variability in matrix- or receptor- binding was also neglected.

As in our previous compartmental models [54], [67], surface receptors were present only in pre-associated homodimeric form, i.e., the biophysical dynamics of VEGF-induced receptor dimerization and VEGFR1-VEGFR2 heterodimers were both ignored, for the lack of *in vivo* validation despite *in vitro* [68], [69] and theoretical evidence [70].

Although *in vitro* studies had found sVEGFR1, in both monomeric and dimeric forms, to be able to form complexes with VEGF in significant amounts in HUVEC-conditioned media [8], [10], there is currently no *in vivo* data on their relative quantities or the dimerization mechanism (whether some sVEGFR1 are secreted as homodimers or whether dimerization is ligand-dependent). Thus in parallel to our surface receptors, sVEGFR1 was modeled as a uniform species of pre-associated homodimers as a first approximation. Furthermore, sVEGFR1-heterodimerization with surface VEGFRs (i.e., the association between monomeric sVEGFR1 and surface VEGFR monomers) was neglected in this first study to independently investigate sVEGFR1's VEGF-trapping function (Fig. 1C: interactions ‘T1-3’).

### 2. Molecular Interactions & Transport Equations

In the mathematical formulation of our model, a system of 61 coupled nonlinear ordinary differential equations (ODEs) was used to describe the temporal dependence of each molecular species' tissue and blood concentrations on their binding interactions and transport processes. Protein concentrations, when expressed in the notations of [*X*]*_j_* and [*X*]*_B_*, as in the ODEs below, are in units of “moles/cm^3^ of tissue *j*” and “moles/cm^3^ of blood” respectively. Elsewhere, separate notations [*X*]*_IS,j_* and [*X*]*_pl_* refer to these concentrations in units of M, as converted to their relevant fluid volume basis, i.e., “moles/L of available interstitial fluid in tissue *j*” and “moles/L of plasma” respectively. The “available” interstitial fluid volume excludes all interstitial fluid spaces that are inaccessible to molecules, including isolated pores (e.g., fluid spaces trapped during matrix deposition) and steric exclusion spaces near solid surfaces (e.g. bound water) [59], [71]. Conversions between the two sets of notations are as follows:
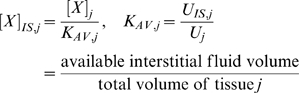


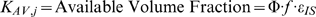


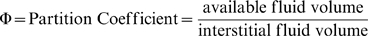


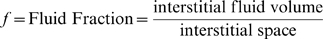


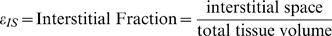
and
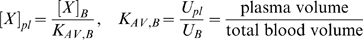



### 2.1 Tissue Equations

Each tissue compartment *j* = {*N* for Normal; *D* for Calf} contained 28 molecular species whose governing equations were as follows:

#### i. Interstitial Matrix

The following 9 equations describe the occupancies of VEGF- and sVEGFR1-binding sites in the interstitial matrix. Here, [*M*] denotes the concentration of matrix binding sites in the ECM, EBM, or PBM as specified in subscripts; [*V*_165_] the interstitial concentration of free VEGF_165_; [*sR_1_*] the interstitial concentration of free sVEGFR1; *k_on_* (moles/cm^3^ tissue/s) and *k_off_* (s^−1^) are the kinetic rates for binding and unbinding respectively. The current model assumed the subsequent binding of free VEGF_165_ onto matrix-bound sVEGFR1 or of free sVEGFR1 onto matrix-bound VEGF_165_ to be negligible; no experimental data on these spatial configurations were available. The unconfirmed binding of VEGF-sVEGFR1 complexes directly onto unoccupied matrix sites was also neglected.
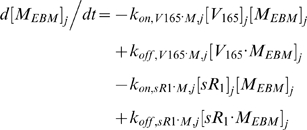


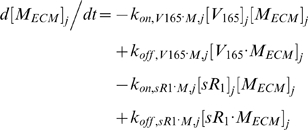


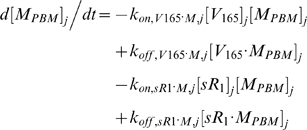


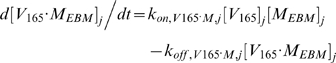


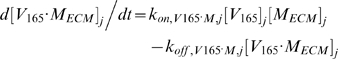


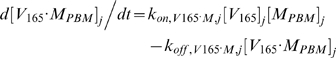


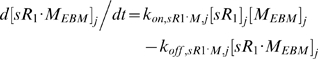


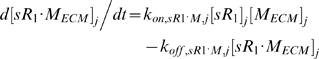


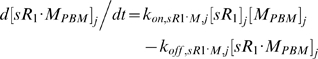



#### ii. Abluminal Endothelial Cell Surface

The next 14 equations describe the ligand-binding and co-receptor-coupling status of abluminal endothelial surface receptors. Notation for the densities of these surface species are: [*R*_1_], [*R*_2_] and [*N*_1_] for unoccupied VEGFR1, VEGFR2 and NRP1 respectively; [*VR*] and [*VN*] for VEGF-bound VEGFRs and NRP1 respectively; [*RVN*] and [*VRN*] for NRP1-coupled VEGF-ligated VEGFRs. *k_c_* (moles/cm^3^ tissue/s) denotes the coupling rate between a VEGFR and the co-receptor NRP1; *k_dissoc_* (s^−1^) denotes the direct decoupling of VEGFR1 and NRP1. *k_int_* (s^−1^) is the internalization rate of free or bound receptors; while *s_R_* is the insertion rate of free receptors back into the cell membrane. In this study, *k_int_* was assumed to be identical for all free receptors and complexes, though there is possibility for future investigation of differential internalization rates for phosphorylated and unphosphorylated receptors. There was also no feedback regulation of receptor expression as a function of VEGF signaling: i.e., constant total (free + occupied) receptor densities were maintained by defining *s_R_* = *k_int_*·[*R*_total_]. Curly brackets contain the terms referring to the direct binding interaction between VEGF_121_ and NRP1, which were turned off (*k_on_* = 0) in the base case and tested during sensitivity analyses.
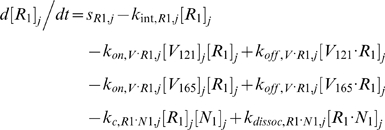


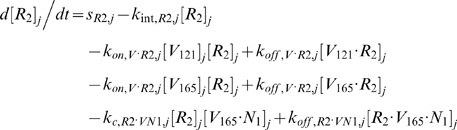


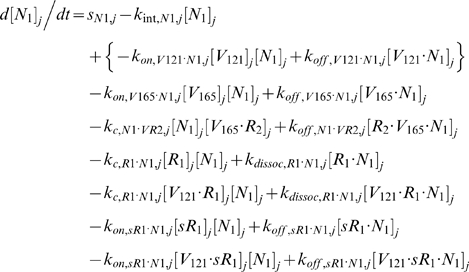


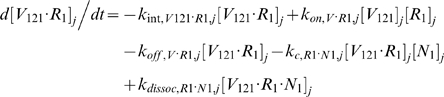


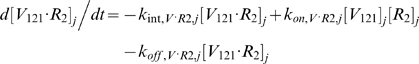


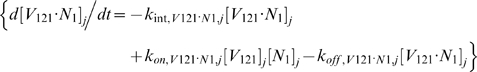


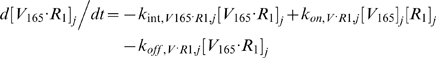


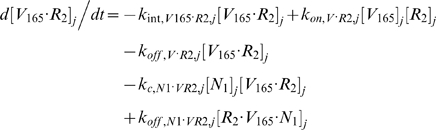


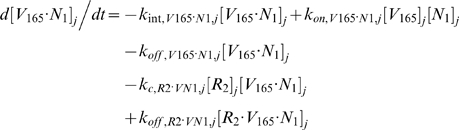


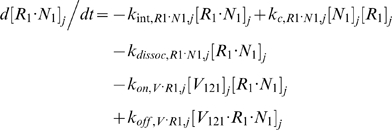


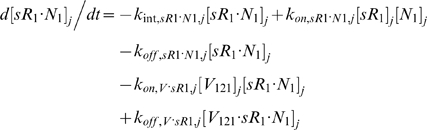


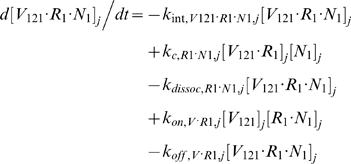


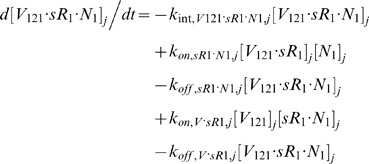


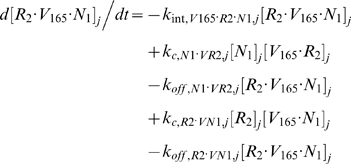



#### iii. Interstitial Fluid

The next 5 equations govern the soluble species of the interstitial fluid, including the complexes formed between the VEGF isoforms and sVEGFR1, the concentrations of which are denoted [*V*·*sR_1_*].
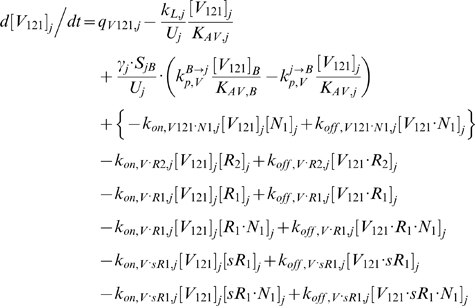


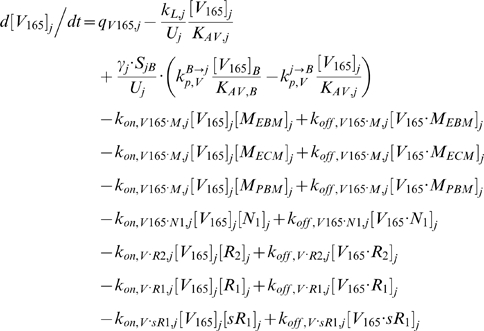


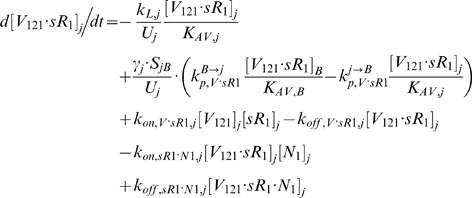


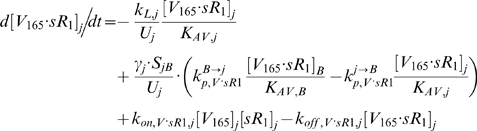


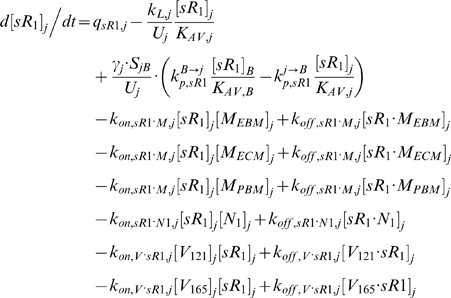



Free VEGF isoforms were secreted at constant rates of *q_V_* (moles/cm^3^ tissue/s) from myocytes; while free sVEGFR1 was secreted abluminally by endothelial cells at a constant rate of *q_sR1_* (moles/cm^3^ tissue/s). The current implementation assumed no time-dependent feedback regulation on secretion rates as a function of VEGFR phosphorylation or internalization. In this study, the cell sources noted above were relevant only for unit conversion of the secretion rates to a “per cell” basis; i.e., the *q_V_* and *q_sR1_* terms would have included endothelial or non-myocyte sources of VEGF and parenchymal sources of sVEGFR1 if these were significant *in vivo*.

All interstitial soluble species were subjected to lymphatic drainage into the blood at a rate of *k_L_* (cm^3^/s), as well as bidirectional (*B→j* or *j→B*) vascular permeability flow at rates of *k_P_* (cm/s). *S_jB_* (cm^2^), which denotes the total abluminal endothelial surface area exposed to the interstitial space of tissue *j*, and γ, which is the endothelial surface area recruitment factor (see Methods section 3.3i), along with *U_j_* and *K_AV_*, were geometric conversion factors that restrict the volumes of interstitial fluid and plasma accessible to macromolecular exchange.

### 2.2. Blood Equations

The last 5 equations described the blood populations of the soluble species. There were no source terms in the blood for VEGF or sVEGFR1 (q_V,B_ = q_sR1,B_ = 0), although luminal secretion from the endothelium or *in situ* release from formed elements (e.g., platelets, monocytes [17]) could be explored in future implementations. In addition to the lymphatic and vascular permeability flows, the soluble species in plasma were subjected to a third transport process – direct clearance at a rate of *k_CL_* (s^−1^).
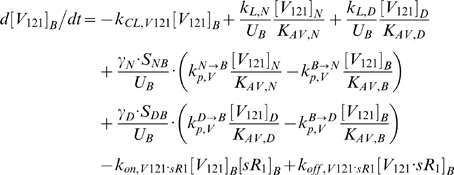


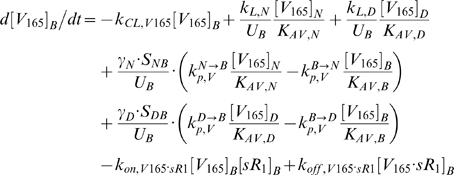


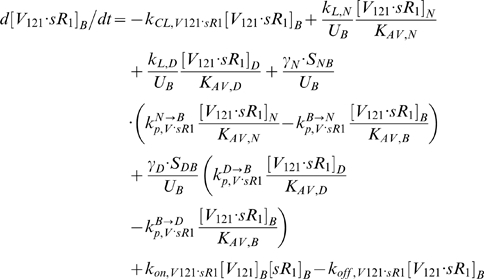


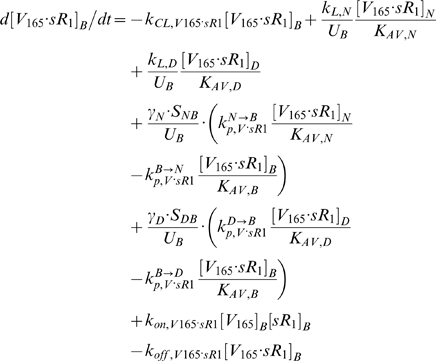


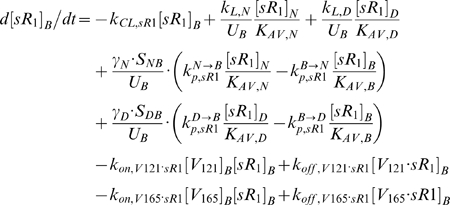



### 3. Model Parameterization for a Healthy Human

This section describes the parameterization of compartmental geometry, binding kinetic rates, biotransport rates, and protein expression levels necessary to solve the model system of ODEs.

### 3.1. Geometry

#### i. Healthy Calf Muscle Compartment

The healthy calf compartment was modeled with a control volume of 868 cm^3^ (unilateral gastrocnemius and soleus muscles) [72], and with the following histological parameters taken from gastrocnemius data of a typical sedentary healthy human, summarized in Table 1.

**Table 1:** Geometric Parameterization of Histological Cross-Sections.

Tissue Compartment	Units	Normal (Rest of Body)		Healthy Calf	
Reference Muscle		Vastus lateralis		Gastrocnemius	
		Value	Ref	Value	Ref
**Compartment Volume**	cm^3^	60453	* [87], [164]	868	† [72]
**Individual Muscle Fibre**					
Diameter	µm	71	*	73	*
Perimeter correction factor	-	1.14	[54]	1.14	[54]
Perimeter	µm	253	*	261	*
FCSA	µm^2^	3904	[88]	4173	[73]
Myonuclei density	mm^−1^	120	[54]	150	[75]
MDSA	µm^2^/MD	2104	*	1740	*
**Muscle Fibre Space**					
Muscle Fibre Density	fibers/mm^2^ tissue	242	*	199	*
FSAV	cm^2^/cm^3^ tissue	611	*	520	*
Muscle Fibre Space Volume Fraction	cm^3^/cm^3^ tissue	94.4%	*	83.1%	*
**Individual Capillary**					
Luminal Diameter	µm	4.86	[89]	3.97	*
Endothelium Thickness	µm	0.77	*	0.78	*
Abluminal Diameter	µm	6.39	[89]	5.53	*
Perimeter correction factor	-	1.1	[54]	1.1	[54]
Abluminal Perimeter	µm	22.1	*	19.1	*
CCSA	µm^2^	32.1	*	24.0	*
i. Lumen CSA	µm^2^	18.6	*	12.4	[76]
ii. Endothelium CSA	µm^2^	13.5	*	11.6	[76]
ECSA (abluminal)	µm^2^/EC	1000	[54]	1000	[54]
**Capillary Space**					
Capillary∶Fiber Ratio	-	1.36	[88]	1.16	[73]
Capillary Density	capillaries/mm^2^ tissue	329	[88]	231	[73]
ESAV (abluminal)	cm^2^/cm^3^ tissue	73	*	44	*
Capillary Space Volume Fraction	cm^3^/cm^3^ tissue	1.1%	*	0.6%	*
i. Endothelium Space	cm^3^/cm^3^ tissue	0.4%	*	0.3%	*
ii. Vascular Space	cm^3^/cm^3^ tissue	0.6%	*	0.3%	*
**Interstitial Space**					
IS Volume Fraction	cm^3^/cm^3^ tissue	4.5%	*	16.3%	*
IF Volume Fraction	cm^3^/cm^3^ tissue	3.7%	*	13.7%	*
Available IF Volume Fraction	cm^3^/cm^3^ tissue	3%		11%	
**i. Extracellular Matrix (ECM)**					
ECM Volume	cm^3^/cm^3^ tissue	3.9%	*	14.9%	*
	cm^3^/cm^3^ IS	86.72%	*	91.24%	*
Solid Fraction ††	cm^3^/cm^3^ ECM	13.40%	[83]	13.40%	[83]
Fluid Volume in ECM	cm^3^/cm^3^ tissue	3.38%	*	12.92%	*
	cm^3^/cm^3^ IF	91.13%	*	94.25%	*
Available Fluid Volume in ECM	cm^3^/cm^3^ tissue	2.87%	*	10.98%	*
**ii. Endothelial Basement Membrane (EBM)**					
Thickness	nm	87.5	[90], [91]	254	[79]–[82]
EBM Volume	cm^3^/cm^3^ tissue	0.06%	*	0.11%	*
	cm^3^/cm^3^ IS	1.41%	*	0.69%	*
Solid Fraction ††	cm^3^/cm^3^ EBM	45%	[83]	45%	[83]
Fluid Volume in EBM	cm^3^/cm^3^ tissue	0.03%	*	0.06%	*
	cm^3^/cm^3^ IF	0.94%	*	0.45%	*
Available Fluid Volume in EBM	cm^3^/cm^3^ tissue	0.01%	‡	0.02%	‡
**iii. Parenchymal Basement Membrane (PBM)**					
Thickness	nm	87.5	[80]	254	[80]
PBM Volume	cm^3^/cm^3^ tissue	0.53%	*	1.32%	*
	cm^3^/cm^3^ IS	11.87%	*	8.07%	*
Solid Fraction ††	cm^3^/cm^3^ SBM	45%	[83]	45%	[83]
Fluid Volume in PBM	cm^3^/cm^3^ tissue	0.29%	*	0.73%	*
	cm^3^/cm^3^ IF	7.92%	*	5.30%	*
Available Fluid Volume in PBM	cm^3^/cm^3^ tissue	0.10%	‡	0.24%	‡

FCSA = fiber cross-sectional area.

MDSA = myonuclear domain surface area.

FSAV = total muscle fibre surface area per tissue volume.

CCSA = capillary cross-sectional area (EC+lumen).

ECSA = endothelial cell surface area.

ESAV = total endothelium surface area per tissue volume.

IS = interstitial space.

IF = interstitial fluid.

* calculated.

† = combined volume of lateral gastrocnemius, medial gastrocnemius, and soleus muscles (unilateral calf).

†† = approximated by collagen content.

‡ calculated for average partition coefficients of 0.85 in ECM and 0.325 in BM (see Table 1b).

From the measured fiber cross-sectional area (FCSA) of 4173 µm^2^ [73], the single muscle fiber was calculated to have a diameter of 73 µm and perimeter of 261 µm (non-cylindrical correction factor of 1.14) [54]. The myonuclear domain (MD) defines the cytoplasmic volume per nucleus within a multinucleated myocyte [74]. Assuming the gastrocnemius to have the same linear myonuclei density of 150 nuclei/mm as that measured from the soleus [75], the MD surface area was then calculated to be 1740 µm^2^/MD. Muscle fiber density (FD) was determined to be 199 fibers/mm^2^ tissue based on a reported capillary density (CD) of 231 capillaries/mm^2^ tissue and a capillary-to-fiber ratio (CF) of 1.16 [73]. This FD yielded a total muscle fiber surface area per unit volume of tissue (FSAV) of 520 cm^2^/cm^3^ tissue and a total muscle fiber volume fraction of 0.831 cm^3^/cm^3^ tissue.

Capillaries of the lower leg were shown to have mean lumen, endothelium and total cross-sectional areas of 12.4, 11.6 and 24.0 µm^2^ respectively [76]. The corresponding luminal and abluminal capillary diameters were calculated to be 3.97 and 5.53 µm respectively, with an endothelium thickness of about 0.78 µm. A capillary perimeter correction factor of 1.1 [54] was assumed to give approximate luminal and abluminal perimeters of 13.7 and 19.1 µm respectively. Together with the mentioned CD, these values yielded a total capillary volume fraction of 0.006 cm^3^/cm^3^ tissue, 0.003 cm^3^/cm^3^ tissue of which was vascular space. The abluminal endothelial cell surface area (ECSA) was taken as 1000 µm^2^ per endothelial cell, averaged from hamster arteriolar [77] and dog aortic endothelial cells [78]; while the total abluminal endothelial surface area per tissue volume (ESAV) available for ligand-receptor binding came to 44 cm^2^/cm^3^ tissue.

As a result, the interstitial space (IS) left between muscle fibers and capillaries had a volume fraction of 0.163 cm^3^/cm^3^ tissue. The healthy calf IS volume comprised: 0.69% endothelial basement membrane (EBM) based on an average thickness of 254 nm [79]–[82]; 8.07% parenchymal basement membrane (PBM) assuming the same thickness as the EBM; and 91.24% extracellular matrix (ECM). Assuming collagen to be the major component in the IS by volume, the solid fraction of the ECM, EBM, PBM were taken as 13.4%, 45% and 45% respectively (dividing 14.175% collagen content in interstitial space [83] between BM and ECM volumes such that collagen density of BMs was triple that of ECM). The resulting fluid fraction of the interstitium – 0.137 cm^3^/cm^3^ tissue, consistent with measured range of 8 to 11% [84] – was in turn only fractionally available to permeable molecules depending on their partition coefficients (Φ). Species-specific Φ (Table 2) were extrapolated from theoretical calibration curves for dextran permeating through networks [85], using the appropriate network pore size (66 nm for ECM [83]; 7 nm for BMs [85]) and the corresponding hydraulic Stokes-Einstein radii between linear polydisperse dextran and globular VEGF-related proteins (conversion formulae from [86]). The final available fluid volumes are summarized in Table 1.

**Table 2:** Hydrodynamic Properties of Soluble Species.

	Molecular Weight	Ref	Hydrodynamic (Stokes-Einstein) Radius, a_E_	Ref	Partition Coefficient, Φ		Ref
					ECM^a^	BM^b^	
**VEGF**	46 kDa	[103]	30 Å	[86]	0.9	0.35	[85]
**sVEGFR1**	220 kDa	[20]	56 Å	[86]	0.8	0.3	[85]
**sVEGFR1-VEGF**	230 kDa	[8]	57 Å	[86]	0.8	0.3	[85]

Assumed pore sizes of ^a^ 66 nm ECM[83].

b 7 nm in BM[85].

#### ii. Normal (Rest of the body) Compartment

The entire body of a 70 kg man minus the blood would weigh about 65 kg. As a first approximation, we modeled the entire 65 kg as homogenous skeletal muscle with a density of 1.06 g/cm^3^ [87], giving a volume of 61,321 cm^3^. Subtracting from it the volume of the healthy calf muscle, the “*normal*” compartment representing the “*rest of the body*” was then determined to be 60,453 cm^3^. The following geometry parameters are slightly different from those we used previously [54], as these are taken directly from human vastus lateralis histological data, without the previous fitting requirements to match water spaces (1.4% vascular space and 7% interstitial fluid), nor previous adjustments based on theoretical oxygen consumptions. This was done for consistency with how calf compartment parameters were derived.

Muscle fiber density (FD) was determined to be 242 fibers/mm^2^ tissue based on a reported capillary density (CD) of 329 capillaries/mm^2^ tissue and a capillary-to-fiber ratio (CF) of 1.36 [88]. Muscle fiber cross-sectional area (FCSA) was experimentally measured at 4150±246 µm^2^ [88]. Multiplying this measured average FCSA of 4150 µm^2^ and the calculated FD of 242 fibers/mm^2^ would have produced a theoretical impossibility – 1.0043 mm^2^/mm^2^ – reflecting overestimations in FCSA and/or FD. Thus the vastus lateralis FCSA was taken instead at one standard error below mean, i.e., 3904 µm^2^. From this conservative measurement of FCSA, the single muscle fiber was calculated to have a diameter of 71 µm and perimeter of 253 µm using a correction factor of 1.14 [54]. Keeping the assumed linear myonuclei density of 120 nuclei/mm [54], the surface area of the myonuclear domain (MD) was then recalculated to be 2104 µm^2^/MD. The new FD also yielded a total FSAV of 611 cm^2^/cm^3^ tissue and a total muscle fiber volume fraction of 0.944 cm^3^/cm^3^ tissue.

The luminal and abluminal diameters of individual capillaries were taken from measurements in the vastus lateralis as 4.86 and 6.39 µm respectively [89]. From these, the endothelium thickness was calculated to be 0.77 µm, while the lumen and endothelium CSA were calculated to be 18.6 and 13.5 µm^2^ respectively, which agree with histological measurements from the mid-thigh (10.8±7.4 and 11.4±6.7 µm^2^) [76]. Assuming the same capillary perimeter correction factor of 1.1 [54], the luminal and abluminal perimeters were then 16.8 and 22.1 µm respectively. Together with the mentioned CD, these yielded a total capillary volume fraction of 0.011 cm^3^/cm^3^ tissue, 0.006 cm^3^/cm^3^ tissue of which was vascular space. Though the abluminal ECSA was still 1000 µm^2^/EC [54], a higher CD compared to the calf contributed to a higher abluminal endothelial surface area per tissue volume (ESAV) of 73 cm^2^/cm^3^ tissue in the normal compartment.

Accordingly, the IS remaining between muscle fibers and capillaries had a volume fraction of 0.045 cm^3^/cm^3^ tissue. The normal compartment IS comprised: 1.41% EBM based on an average thickness of 87.5 nm [90], [91] (instead of the 43 nm previously taken [54] from extreme athletes to avoid extra exercise-trained effects [92]); 11.87% PBM assuming the same thickness as the EBM; and 86.72% ECM. As summarized in the Table 1, the available fluid fractions of each IS volume were calculated using the same solid fractions and Φ's as in healthy gastrocnemius.

#### iii. Blood Compartment

Finally, the blood compartment was modeled with a volume of 5L, 60% of which was plasma [93]. Ultrastructures that were not explicitly modeled include the glycocalyx/cell-free layer or interendothelial clefts, the effects of which were incorporated within the transport parameters described below. Formed elements in the blood (e.g., platelets and leukocytes) were also ignored but could be considered as binding sites and/or sources for the soluble species in future model extensions.

### 3.2. Binding and Coupling Kinetics

The control values for all binding and coupling kinetic rates (Table 3), except for the new interactions involving sVEGFR1, were kept identical to those from our previous single-compartment skeletal muscle model [54]. However, sensitivity analyses were performed to explore the following wide ranges cited in literature. VEGF affinity of VEGFR1 and VEGFR2 varied from *K_d_* = 1–130 pM and 72–760 pM depending on culture cell type (e.g., HUVECs vs. tPAEs vs. HUCMECs) [94]–[103]. VEGF_165_ affinity of NRP1s additionally varied with assay type: *K_d_* = 200–320 pM by cell-based Scatchard [100], [104] vs. 25–120 nM by cell-free BIAcore surface plasmon resonance analysis [14], [105]. In fact, against general consensus that VEGF_121_ does not bind NRP1, Pan *et al.* recently observed such binding at *K_d_* = 220 nM (BIAcore), albeit at a lower affinity than the 120 nM reported for VEGF_165_ and NRP1 in the same study [105]. This finding coincided with recent observations that in addition to exon 7 of VEGF (which VEGF_165_ possesses while VEGF_121_ does not) that was traditionally associated with NRP-binding, exon 8 (which all VEGF isoforms have some form of) might also confer NRP-affinity [106]. We thus tested both scenarios: adding a new interaction between VEGF_121_ and NRP1 at a dissociation constant 1.83 times higher than our control *K_d_* for VEGF_165_ and NRP1 (to match the *K_d_* ratio measured by Pan *et al.*); and setting NRP1 affinities for both VEGF isoforms to the exact values as Pan *et al.* reported. Lastly, we explored lower VEGF-binding affinities for matrix sites, considering that our control value of VEGF_165_'s *K_d_* for matrix sites was estimated from that of FGF-2 [107], in light of indication that VEGF_165_'s *K_d_* for modified heparin could be ∼6–7 times higher than that of FGF-2 [108], [109].

**Table 3:** Kinetic Parameters of the VEGF system (Control Values).

Compartment	In Vitro Measurements		Simulation Control Values (In Vivo Conversions)			
	Culture Medium		Blood	Normal	Healthy Calf	Unit
	Value	Unit	Value	Value	Value	
**VEGF binding to VEGFR1**						
k_on_	3·10^7^	M^−1^ s^−1^	N/A	10^12^	2.73·10^11^	(mol/cm^3^ tissue)^−1^s^−1^
k_off_	10^−3^	s^−1^				
K_d_	3.33·10^−11^	M	N/A	10^−15^	3.67·10^−15^	mol/cm^3^ tissue
**VEGF binding to VEGFR2**						
k_on_	10^7^	M^−1^ s^−1^	N/A	3.33·10^11^	9.09·10^10^	(mol/cm^3^ tissue)^−1^s^−1^
k_off_	10^−3^	s^−1^				
K_d_	10^−10^	M	N/A	3·10^−15^	1.1·10^−14^	mol/cm^3^ tissue
**VEGF_121_ binding to NRP1**						
k_on_	0	M^−1^ s^−1^				
k_off_	0	s^−1^				
K_d_	N/A	M				
**VEGF_165_ binding to NRP1**						
k_on_	3.125·10^6^	M^−1^ s^−1^	N/A	1.04·10^11^	2.84·10^10^	(mol/cm^3^ tissue)^−1^s^−1^
k_off_	10^−3^	s^−1^				
K_d_	3.2·10^−10^	M	N/A	9.6·10^−15^	3.52·10^−14^	mol/cm^3^ tissue
**VEGF_165_ binding to GAG**						
k_on_	4.2·10^5^	M^−1^ s^−1^	N/A	1.4·10^10^	3.82·10^9^	(mol/cm^3^ tissue)^−1^s^−1^
k_off_	10^−2^	s^−1^				
K_d_	2.38·10^−8^	M	N/A	7.14·10^−13^	2.62·10^−12^	mol/cm^3^ tissue
**VEGFR1 coupling to NRP1**						
k_c_	10^14^	(mol/cm^2^)^−1^s^−1^	N/A	1.37·10^12^	2.27·10^12^	(mol/cm^3^ tissue)^−1^s^−1^
k_dissoc_	10^−2^	s^−1^				
**VEGF·VEGFR2 coupling to NRP1**						
k_c_	3.1·10^13^	(mol/cm^2^)^−1^s^−1^	N/A	4.25·10^11^	7.05·10^11^	(mol/cm^3^ tissue)^−1^s^−1^
k_off_	10^−3^	s^−1^				
**VEGF·NRP1 coupling to VEGFR2**						
k_c_	10^14^	(mol/cm^2^)^−1^s^−1^	N/A	1.37·10^12^	2.27·10^12^	(mol/cm^3^ tissue)^−1^s^−1^
k_off_	10^−3^	s^−1^				
**VEGF binding to sVEGFR1** *						
k_on_	3·10^7^	M^−1^ s^−1^	5·10^10^	10^12^	2.73·10^11^	(mol/cm^3^ tissue)^−1^s^−1^
k_off_	10^−3^	s^−1^				
K_d_	3.33·10^−11^	M	2·10^−14^	10^−15^	3.67·10^−15^	mol/cm^3^ tissue
**sVEGFR1 coupling to GAG** †						
k_on_	4.2·10^5^	M^−1^ s^−1^	7·10^8^	1.4·10^10^	3.82·10^9^	(mol/cm^3^ tissue)^−1^s^−1^
k_off_	10^−2^	s^−1^				
K_d_	2.38·10^−8^	M	1.43·10^−11^	7.14·10^−13^	2.62·10^−12^	mol/cm^3^ tissue
**sVEGFR1 binding to NRP1** ‡						
k_on_	5.56·10^6^	M^−1^ s^−1^	9.26·10^9^	1.85·10^11^	5.05·10^10^	(mol/cm^3^ tissue)^−1^s^−1^
k_off_	10^−2^	s^−1^				
**Receptor Internalization**						
k_int,R_ (free receptors)	2.8·10^−4^	s^−1^				
k_int,C_ (bound receptors)	2.8·10^−4^	s^−1^				

For in vitro values: * assumed same as VEGF binding to VEGFR1 [95].

† assumed same as VEGF_165_ binding to GAG.

‡ assumed same k_off_ as VEGFR1 coupling to NRP1, with k_on_ calculated to match K_d_ from literature [14] ; the rest were taken from [54].

Conversions for in vivo values were based on geometry parameters from Table 1:
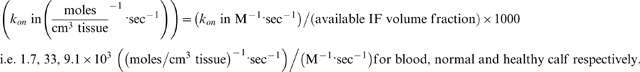


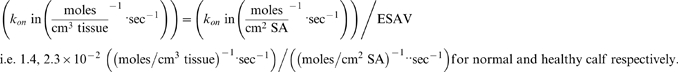

Regarding the new sVEGFR1 interactions with VEGF, matrix sites and NRP1, the following assumptions were made for their kinetic rates. *In vitro* experiments have characterized the VEGF-affinity of baculovirus-expressed and HUVEC-derived human sFlt-1 at *K_d_*∼20 and 10 pM respectively [10], [20]. Given the *in vitro* evidence that the soluble and full-length VEGFR1's have similar affinities to VEGF [95], we assumed in our model a *K_d_* of 33 pM for VEGF-sVEGFR1 binding, same as that previously used [54] for VEGF-VEGFR1 binding. To the best of our knowledge, sVEGFR1-binding affinities of various HSPGs have not been characterized. Despite the possible heparin-binding domain differences between VEGF and sVEGFR1, we took the effective *K_d_* = 23.8 nM previously used [54] for VEGF binding to matrix-sites (ensemble of various HSPGs) as the control value of our *K_d_* for sVEGFR1 binding to matrix-sites. BIAcore binding analysis of Flt-1 extracellular domains (ECDs) to immobilized NRP-1 showed a *K_d_* of 1.8 nM [14]. Approximating sVEGFR1 as Flt-1 ECDs, we modeled sVEGFR1's affinity to NRP-1 with a *K_d_* of 1.8 nM, subsequently calculating the *k_on_* based on the same *k_off_* as full-length VEGFR1's dissociation from NRP1 [54].

### 3.3. Transport

#### i. Macromolecular Vascular Permeability

Here, the vascular permeability rates (*k_P_*) characterized the microvasculatures' baseline (supine posture) capacity in allowing macromolecular transport across the endothelium, as in our previous study [59]. A new parameter representing endothelial surface area recruitment (*γ*) was introduced to account for changes in the degree of perfusion within capillary beds as occurs during physiological redistribution of blood – e.g., a *γ*<1 would represent effectively reduced transendothelial transport due to a larger fraction of unperfused or non-distended capillaries.

In determining the basal vascular permeability rates for the soluble species, their molecular weights (MW) were first converted into their Stokes-Einstein radii (a_E_) based on the empirical relation for globular proteins, a_E_ = 0.483(MW)^0.386^ [86] (Table 2). The corresponding permeability-surface area product (PS) were then determined from a set of theoretical curves for PS vs. a_E_ [110]. Finally, assuming a capillary surface area (S) of 70 cm^2^/g muscle tissue [111], the molecular species-specific basal permeabilities (*k_p_* = PS/S) were obtained (Table 4).

**Table 4 pone-0005108-t004:** Transport Parameters: Basal Vascular Permeability and Plasma Clearance Rates.

	Basal Permeability Rates* (bidirectional)		Clearance Rate from Blood	
	*k_P_* [cm/s]	Ref	*k_C_* [s^−1^]	Ref
**VEGF**	4.29·10^−8^	[110], [111]	1.08·10^−3^	[120]
**sVEGFR1**	1.86·10^−8^	[110], [111]	5·10^−6^	Estimated from VEGF-traps [121], [122], [165]
**sVEGFR1-VEGF**	1.86·10^−8^	[110], [111]	5·10^−6^	Assumed same as sVEGFR1 based on hydrodynamic size

* Calibrated based on hydrodynamic sizes from Table 1b.

The posture and activity-dependent surface area recruitment factors (Table 5) were approximated as follows. The effective vascular permeabilities of the normal “rest of the body” compartment were maintained at basal levels (γ = 1) in both supine (lying down) and dependent (sitting/standing) positions, assuming there to be little change after averaging the pressure/perfusion changes to tissue masses above and below the heart level. For the “healthy calf” compartment, however, the venoarterial reflex – which normally decreases foot perfusion in response to moving from a supine to dependent position in compensation to the gravitational hydrostatic hypertension – was taken into consideration. Specifically, foot skin blood perfusion in normal subjects has been found to decrease from 7.8±2.2 mL/min/100 g in the supine position to 2.8±0.6 mL/min/100 g in the dependent position [112]. We thus assumed the “healthy calf” compartment to take on the basal permeabilities when at rest (γ = 1 for supine) and at 3/8 of the basal levels when standing (γ = 0.375 for dependent). Exercise, on the other hand, has been shown to increase capillary surface area available for perfusion up to 2× and 3× resting levels in rats and humans respectively [113], [114]; we thus allowed γ = 3 for an exercising calf.

**Table 5 pone-0005108-t005:** Transport Parameters: Surface Area Recruitment Factor for Effective Vascular Permeability Rates.

Surface Area Recruitment Factor, *γ_j_*	j = Normal (Rest of Body)		j = Healthy Calf	
	Value	Ref	Value	Ref
**Supine (control)**	1	basal	1	basal
**Dependent**	1	basal	0.375	VAR [112]
**Exercise (leg)**	1	basal	3	[113], [114]

VAR = venoarterial reflex.

#### ii. Lymphatic Drainage

In mice quadriceps femoris skeletal muscle, capillary-sized lymphatic vessels were found next to blood capillaries between muscle fibers, but at a much lower density [115]. Hence the small volume of the lymphatic capillary space was not explicitly partitioned within our tissue geometries (Fig. 1A). There is no macromolecular size-dependence in the filling of the initial lymphatics (unlike macromolecular permeability through blood capillaries) [62], thus protein concentrations drained through the lymphatics were modeled as continuous with those in the available interstitial fluid. Moreover, fluid transfer through the lymphatics was assumed to have negligible effects on volume and protein concentration changes in interstitial fluid and plasma. This was consistent with our representation of vascular permeability where only macromolecular but not hydraulic transport was represented in the model. The following parameterization is summarized in Table 6.

**Table 6 pone-0005108-t006:** Transport Parameters – Lymphatic Flow Rates.

Tissue Compartment	“Normal”	“Calf”	Unit	Ref
	Value	Value		
Lymphatically-drained tissue mass	50.02	0.92	kg	a ^,^ b
**Basal Levels**				
Supine; Night/Asleep	2 (1.7–2.5)		µL/h/(g SkM)	[62]
*k_L_*/mass	5.6·10^−7^		cm^3^/s/(g tissue)	c
*k_L_*	0.028	0.0005	cm^3^/s	
Supine; Day/Awake (Simulation Control) *k_L_*	0.14	0.0026	cm^3^/s	∼5× asleep [118]
**Active Levels**				
Steady Exercise *k_L_*	0.28	0.0051	cm^3^/s	∼2× awake [64], [119]
Peak Exercise *k_L_*	0.70	0.013	cm^3^/s	∼5× awake [64]

Conversions are based on: ^a^ the mass composition of a 70-kg man as 5-kg blood, ∼14 kg non-lymphatically drained mass, ∼51 kg lymphatically drained mass [60], [116], [117].

b healthy calf volume from Table 1 with 1.06 g/cm^3^ [72], [87].

c assuming all tissues to have the skeletal muscle lymph flow rate.

To approximate the total mass of body tissue subjected to lymphatic drainage, the masses of organs with no lymphatic capillaries (entire central nervous system, lens, cornea, bone, bone marrow, epidermis [60], [116]; ∼14 kg [117]) and the mass of blood (∼5 kg) were first subtracted from 70-kg total body mass. The resulting 51 kg of body tissue drained by lymphatics was then divided between the ∼0.92 kg “calf” [72], [87] and the ∼50.08 kg “normal” compartments. The night-time (asleep; supine position) lymph flow per gram of human skeletal muscle was measured to be ∼2 µL/h/g skeletal muscle or 5.6×10^−7^ cm^3^/s/g tissue [62]. Thus the night-time lymph flow for the “calf” compartment was determined to be 0.0005 cm^3^/s. Approximating all other lymphatically-drained organs to have the skeletal muscle lymph flow rate yielded a lymph flow of 0.028 cm^3^/s for the “normal” compartment. Awakeness alone (awake; supine posture) was found to increase the average basal lymph flow rates to about 5-fold of night values [118]; these day-time basal values were taken as the control rates for our simulations. Additionally, healthy subjects would experience a range of muscle activity-dependent lymph flow rates through the course of a day. Lymph flow was found to increase 10 to 13-fold from basal night levels during normal daily activities (steady-state standing/walking) [118], [119], and could peak to 5-fold of basal day levels at 1 h post-onset of exercise (running/cycling) [64].

#### iii. Plasma Clearance

The plasma clearance rates (*k_CL_*, in units of s^−1^) or terminal half-lives (*τ* = (ln 2)/*k_CL_*, in units of s) of proteins used in this model (Table 4) represented the non-specific physiological elimination processes that include kidney filtration, liver conjugation, and proteolytic catabolism. Where possible, we distinguished this type of clearance from the early plasma clearance processes such as rapid uptake by various tissue organs (biodistribution phase), which was represented separately in our model by the extravasation of proteins into tissue compartments (*k_P_*) with subsequent receptor-specific sequestration (*k_c_*, *k_on_*) and internalization (*k_int_*). For VEGF in particular, we approximated from Eppler *et al.*'s compartmental model that distinguished between receptor-specific tissue uptake and non-specific elimination components of plasma clearance [120]. Their fitted dose-independent clearance rate for VEGF_165_ was 3.89 h^−1^ or 1.08×10^−3^ s^−1^, corresponding to an effective clearance half-life of ∼10.7 min. Synthetic soluble VEGF-traps that consisted of partial extracellular domains of VEGFR1 (or VEGFR1 and VEGFR2) fused to the Fc region of IgG1 (dimers) were found to have clearance rates in the range of ∼3 to 8×10^−6^ s^−1^ [121], [122]. Thus for endogenous sVEGFR1 and VEGF-sVEGFR1, with no documented clearance rates, we assumed a clearance rate of 5×10^−6^ s^−1^.

### 3.4. Protein Expression Levels

#### i. Interstitial Matrix Binding Sites for VEGF and sVEGFR1

Of the various types of glycosaminoglycans (GAGs) present on proteoglycans, VEGF_165_ interacts predominantly with heparin and heparan sulfates (HS), while considerably less with chondroitin sulfate or dermatan sulfate [123]. As heparin is mostly localized on mast cells [123], we derived our matrix-specific VEGF-binding site densities from only HS-proteoglycans (HSPGs), as summarized in Table 7. Other contributions to VEGF-binding matrix sites from glycoproteins such as fibronectin were ignored due to having concentrations 1–3 orders of magnitude lower than HSPG contributions in the ECM and BM [124]. Cell-surface HSPGs [125], as well as soluble-HSs, soluble-fibronectins and platelets in the plasma [126], [127], were also not explicitly modeled.

**Table 7 pone-0005108-t007:** Total Interstitial VEGF/sVEGFR1-Binding Site Densities, Based on Matrix Heparan Sulfate Proteoglycans (HSPGs).

“Normal” & “Calf” Tissue Compartments	Value	Unit	Ref
**ECM**	[Reference Tissue:	Cultured endothelial cells]	
[FGF-binding HS-sites]_ECM_	1.5·10^12^	available sites/mm^2^ ECM	[166]
Simulation Control	0.75	µM	[107]
[VEGF-binding HS-sites]_ECM_			
Simulation Control	2.15·10^−11^	moles/cm^3^ Normal tissue	
[VEGF-binding HS-sites]_ECM_	8.24·10^−11^	moles/cm^3^ Healthy Calf tissue	
**EBM/PBM**	[Reference Tissue:	EHS sarcoma in control mice]	
[HSPGs]_IS,EHS_	0.49	mg HSPG/g EHS tumor	[128]
	1.13	g HSPG/L of IS_EHS_	a
[HSPGs]_BM,SkM_	2.92	µmoles total HSPG/L of total BM_SkM_	b ^, ^ c
[VEGF-binding HS-sites]_BM_	12.3	µmoles total binding sites/L of total BM_SkM_	d
	21.2	µmoles available binding sites/L of available fluid in BM_SkM_	e
Simulation Control	20	µM	d
[VEGF-binding HS-sites]_BM_			
Simulation Control	2·10^−12^	moles/cm^3^ Normal tissue	
[VEGF-binding HS-sites]_EBM_	4·10^−12^	moles/cm^3^ Healthy Calf tissue	
Simulation Control	2·10^−11^	moles/cm^3^ Normal tissue	
[VEGF-binding HS-sites]_PBM_	4.8·10^−11^	moles/cm^3^ Healthy Calf tissue	

EHS = Engelbreth-Holm-Swarm.

SkM = skeletal muscle. Conversions are based on: ^a^ cell volume fraction of 0.57 in tumor tissue [83].

b average 387-kDa HSPG [see text].

c the IS of EHS tumors is homogenously BM [128].

d ∼4.2 VEGF-binding sites per HSPG.

e 31% (available fluid/fluid volume)_BM_ and 18% (available fluid/total volume)_BM_ based on Table 1 geometry.
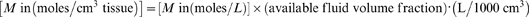


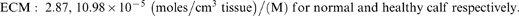


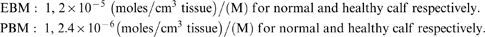

In the ECM, the VEGF-binding matrix-site densities were estimated from FGF-binding sites as in our previous models [54], [107]. In the BMs, we modeled the HSPG content of both tissue compartments based on measurements from Engelbreth-Holm-Swarm (EHS) sarcomas in control (non-diabetic) mice [128]. EHS mouse tumors produce a homogenous interstitial matrix of basement membrane material, making EHS interstitial matrix a typical *in vitro* model for physiological BMs which are usually too thin for content analysis [128], [129]. To convert the HSPG protein weights into molar concentrations, we assumed the average HSPG to weigh 387 kDa – a sum of the 300 kDa protein core weight (averaged over 470-kDa Perlecan [130], 250-kDa Agrin [131], 180-kDa Collagen XVIII PG [131]) and ∼3 HS-chains (averaged 29 kDa [132]) per HSPG. To convert the HSPG content into VEGF-binding site densities, we further assumed an average of 4.2 VEGF-binding sites per HSPG, based on experiments showing: ∼3 HS-chains per HSPG [130], [131]; ∼30% of HS-chains displaying no VEGF_165_ affinity [123]; ≥2 VEGF-binding sequences (“SAS domains”) present within each HS-chain [123], [125].

Due to insufficient literature data on the availability of sVEGFR1-binding sites on HSPGs, we did not separately calculate their corresponding densities and instead assumed that sVEGFR1 would share (competitively) the same matrix sites with VEGF.

#### ii. Endothelial Cell Surface Receptors

For the “normal body” and “healthy calf” compartments, we converted the same VEGFR1 and VEGFR2 protein measurements [133], [134] from human vastus lateralis muscles referenced in our previous models [54] – using our new geometric properties (73 cm^2^/cm^3^ tissue) and dimeric molecular weights of the VEGFRs – to get corresponding density ranges of 60,000–68,000 VEGFR1 and 10,000–14,000 VEGFR2 per endothelial cell. NRP1 protein expression *in vivo* has yet to be documented in literature. As before [54], we interpreted these density ranges as upper bounds for our control values – assumed at 10,000/EC for all VEGFRs – since the measurements did not discriminate between intracellular vs. surface-bound vs. soluble forms of the receptors. There was also evidence suggesting VEGFR2 densities may actually be higher than VEGFR1 densities [19], [96]–[98]. Without definitive quantification, we assumed a 1∶1∶1 ratio of VEGFR1∶VEGFR2∶NRP1 densities at control. Converted values are tabulated in Table 8.

**Table 8:** Total Surface Receptor Densities.

“Normal” & “Calf” Tissue Compartments	Value	Unit	Ref
**VEGFR1**			
Experimental Measurement	1.6–1.8	pg/µg protein	[133], [134]
	0.73–0.82	pmoles/cm^3^ tissue	
	60,000–68,000	#/EC	
Simulation Control	10,000	#/EC	
	1.21·10^−13^ ;	moles/cm^3^ Normal tissue ;	
	7.31·10^−14^	moles/cm^3^ Healthy Calf tissue	
**VEGFR2**			
Experimental Measurement	0.33–0.5	pg/µg protein	[133], [134]
	0.12–0.18	pmoles/cm^3^ tissue	
	10,000–14,000	#/EC	
Simulation Control	10,000	#/EC	
	1.21·10^−13^ ;	moles/cm^3^ Normal tissue ;	
	7.31·10^−14^	moles/cm^3^ Healthy Calf tissue	
**NRP1**			
Experimental Measurement	N/A		
Simulation Control	10,000	#/EC	
	1.21·10^−13^ ;	moles/cm^3^ Normal tissue ;	
	7.31·10^−14^	moles/cm^3^ Healthy Calf tissue	

Experimental measurements from human vastus lateralis muscle. Conversions are based on 155 mg protein/g tissue [167], 1.06 g/cm^3^ tissue [87] , 360-kDa VEGFR1 homodimers [103], 460-kDa VEGFR2 homodimers [103] and microvessel surface area densities (ESAV) from Table 1.
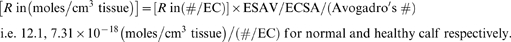

#### iii. Interstitial Free VEGF and sVEGFR1

In healthy humans at rest, the interstitial free VEGF concentration has been measured in vastus lateralis microdialysates to be about 12–50 pg/mL [135], [136], which converts to 0.26–1.1 pM based on 46-kDa VEGF dimers. We thus targeted a range of 0.5–1.0 pM in the “normal body” and “healthy calf” interstitia (Table 9). The basal interstitial sVEGFR1 concentrations have not been reported in the literature. We thus estimated a target range for interstitial sVEGFR1 concentrations – by scaling the target range for interstitial VEGF concentrations to the VEGF∶sVEGFR1 protein weight measurements in tibialis anterior (TA) homogenates in normal mice [19] – at about 0.6–1.2 pM (Table 9). Limitations were noted in extrapolating from homogenate measurements that did not discriminate between interstitial and intracellular proteins. Thus in the case that plasma and interstitial concentrations could not be simultaneously modeled, we opted to fit targeted ranges for plasma concentrations in violation of the interstitial targets.

**Table 9:** Steady-State Interstitial Concentrations of VEGF and sVEGFR1.

“Normal” & “Calf” Tissue Compartments	Value	Unit	Ref
**[VEGF]_IS_**			
Reference Tissues	Human VL microdialysate		
Experimental Range	12–50.4	pg/mL	[135], [136]
	0.26–1.1	pM	a
Simulation Target Range	0.5–1.0	pM	
Achieved @ Healthy Ctrl	10	pM	Fig. 4
**[sR1]_IS_**			
Reference Tissues	Calculated for human VL; Ratios from mice TA homogenates		
Experimental Range	Calculated from (sVEGFR1∶VEGF) _NC, baseline_ = 120∶21 pg/mg protein		[19]
Simulation Target Range	0.6–1.2	pM	b
Achieved @ Healthy Ctrl	36	pM	Fig. 4

Conversions are based on: ^a^ 46-kDa VEGF-dimers [103].

b 220-kDa sVEGFR1-homodimers.

#### iv. Plasma Free VEGF and sVEGFR1

In healthy subjects, measurements of plasma VEGF concentrations generally fell within two ranges: 78–113 pg/mL plasma (∼2 pM) [44]–[46] and 32–41 pg/mL plasma (∼1 pM) [49], [137], [138]. We thus simulated plasma VEGF at 1.5 pM as healthy control (Table 10).

**Table 10:** Steady-State Plasma Concentrations of VEGF and sVEGFR1.

Blood Compartment	Value	Unit	Ref
**[VEGF]_plasma_**			
Experimental Range 1 (healthy controls in PAD studies)	78–113	pg/mL plasma	[44]–[46]
Experimental Range 2 (resting controls in exercise studies)	32–41	pg/mL plasma	[49], [137], [138]
Simulation Target Range	1–2	pM	a
Achieved @ Control	1.5	pM	
**[sR1]_plasma_**			
Experimental Range 1 (higher range of healthy controls in PAD studies)	21–22	ng/mL plasma	[44]–[46]
Experimental Range 2 (lower range of healthy controls in PAD studies)	0.9	ng/mL plasma	[43], [46]
Simulation Target Range	100	pM	b from range 1
Achieved @ Control	100	pM	

Conversions are based on: ^a^ 46-kDa VEGF-dimers [103].

b 220-kDa sVEGFR1-homodimers.

Literature data on plasma sVEGFR1 concentrations were significantly more heterogeneous [39], [40], [42]–[52]. In defining our target ranges, we limited our sources to healthy control data from PAD-specific studies, for consistency in data source compared to our concurrent studies modeling PAD patients. Since most healthy measurements from studies on other atherosclerotic vascular diseases (e.g. coronary artery disease (CAD), diabetes with atherosclerosis [44], [52]) also supported the higher healthy control range of 21–22 ng/mL as reported in PAD studies [44], [45], we simulated plasma sVEGFR1 at ∼100 pM as healthy control (Table 10), such that our “healthy subject” was defined within the same control demographics typically considered in PAD/CAD/diabetic studies.

### 4. Numerical Solutions & Computational Simulations

All simulations and data plots presented in this chapter were performed on the numerical computing platform, MATLAB 7.3.0 R2006b (MathWorks, Natick, MA), and run on a desktop PC. The full system of coupled nonlinear ODEs described above was solved as an initial value problem to steady state using MATLAB's ‘ode15s’ solver routine (a multi-step variable-order stiff problem solver that employs the numerical differentiation formulas scheme), with the relative error tolerance set at 10^−6^ (0.0001% accuracy) and an initial step size of 10^−4^.

The following is an outline of the simulation experiments performed and the organizational structure of the results section below. Results section 1 covers the initial steps of finding the tissue secretion rates necessary to reproduce a healthy control subject's expected ranges of interstitial and plasma concentrations of VEGF and sVEGFR1 (Tables 9 and 10), with all other parameters (geometry, kinetics, transport and protein expression) set at values summarized for a supine healthy subject (Tables 3 to [Table pone-0005108-t004] [Table pone-0005108-t005] [Table pone-0005108-t006] [Table pone-0005108-t007] 8). At the established control settings, a detailed molecular characterization of the healthy model is then given: the steady-state compartmental distributions of total VEGF and sVEGFR1; the tissue-specific profiles of matrix site occupancies and receptor signaling; and the balance of mass flows. Such characterization proved useful in explaining VEGF/sVEGFR1 system responses observed in subsequent parameter sensitivity analyses of: the secretion rates of VEGF and sVEGFR1 (Results section 2); surface receptor densities (section 3); VEGF-affinities of surface receptors (section 4); VEGF-affinities of interstitial matrix sites (section 5); and transport parameters (section 6).

## Results

### 1. Determining basal profiles for a healthy human subject

### 1.1. Targeting experimental plasma concentrations of VEGF and sVEGFR1 as functions of their secretion rates from the “normal” and “calf” compartments

In our stepwise search for the set of secretion rates that could computationally replicate the interstitial and plasma concentrations of VEGF and sVEGFR1 expected from experimental data in our healthy control model, we first established the VEGF concentrations in absence of sVEGFR1, and then introduced sVEGFR1 at modified secretion rates.

**Figure 2:**
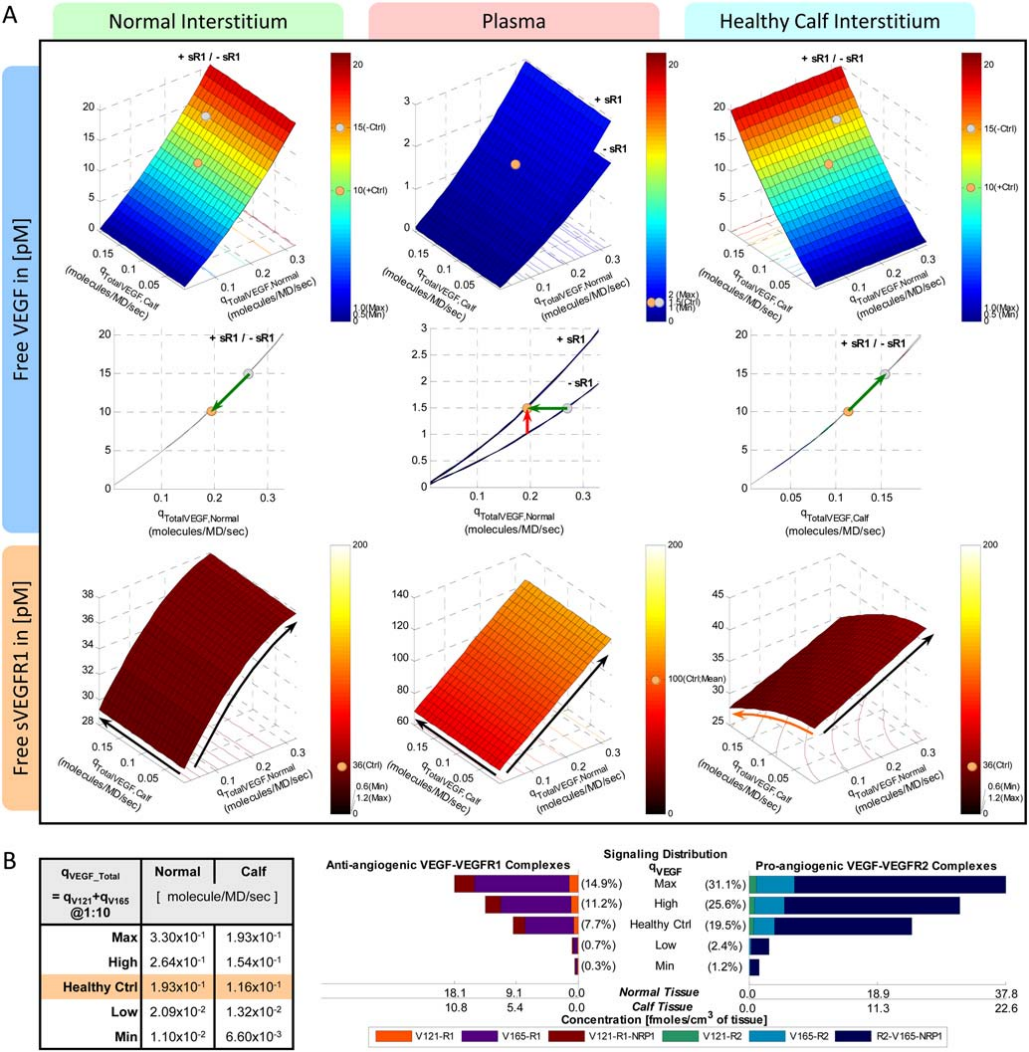
Targeting Control VEGF-Secretion Rates (q_TotalVEGF_) for Basal Profile of Healthy Subject. A. *Steady-state sensitivity of plasma and interstitial concentrations of free VEGF (top and middle rows) and free sVEGFR1 (bottom row) to q_TotalVEGF_ from normal tissue (y-axis) and calf (x-axis*. VEGF isoforms were secreted at a ratio of VEGF_121_∶VEGF_165_ = 1∶10. *Top/middle row:* In absence of sVEGFR1 (q_sR1_ = 0; labelled ‘-sR1’), plasma free VEGF reached the targeted 1.5 pM at the control VEGF-secretion rates of q_V,-Ctrl_ = (q_TotalVEGF,Normal_, q_TotalVEGF,Calf_) = (0.264,0.154) molecule/MD/s. The incorporation of sVEGFR1 expression (q_sR1_ = q_sR1,Ctrl_; labelled ‘+sR1’) raised plasma VEGF significantly (red arrow) but with negligible effects on interstitial VEGF. To keep plasma free VEGF at the targeted 1.5 pM, the control VEGF-secretion rates were redefined (green arrow) to be q_V,+Ctrl_ = (q_TotalVEGF,Normal_, q_TotalVEGF,Calf_) = (0.1925,0.1155) molecule/MD/s. Grey and beige spheres mark the interstitial and plasma VEGF levels reached at q_V,-Ctrl_ and q_V,+Ctrl_ respectively. *Bottom row:* Despite sVEGFR1's role as a VEGF sink, free sVEGFR1 only changed inversely relative to free VEGF changes in the calf interstitum in the direction of increasing q_TotalVEGF,Calf_. Orange/black arrows indicate inverse/direct relation between sVEGFR1 concentrations and VEGF-secretion rates. B. *Density of VEGF-VEGFR complexes changed in proportion to interstitial free VEGF levels with increasing q_TotalVEGF_.* Bracketed percentages are VEGF-bound fractional occupancies of total VEGFR, averaged (range<0.3%) between normal and calf compartments. In figure: ‘+’ = control; ‘max’ and ‘min’ bound targeted ranges; ‘MD’ = myonuclear domain; ‘V121’ = VEGF_121_; ‘V165’ = VEGF_165_; ‘R1’ = VEGFR1; ‘sR1’ = sVEGFR1; ‘R2’ = VEGFR2.

In the absence of sVEGFR1 expressions (q_sR1_ = 0), the steady-state plasma and interstitial free VEGF concentrations ([V]*_pl_*, [V]*_IS_*) were mapped out as functions of total VEGF-secretion rates from the normal tissue and calf compartments (q_TotalVEGF,Normal_ and q_TotalVEGF,Calf_), where the individual isoforms VEGF_121_ and VEGF_165_ were secreted at a ratio of 1∶10 (Fig. 2A: top row, ‘-sR1’). Where [V]*_pl_* was within the experimental ranges of 1–2 pM, [V]*_IS_* were predicted to be ∼10-fold higher, in contradiction of microdialysis measurements of [V]*_IS_* at around 1 pM. Therefore, in the absence of sVEGFR1 expression, the control VEGF-secretion rates necessary to achieve the targeted [V]*_pl_* of 1.5 pM, as well as symmetric [V]*_IS_* (i.e., 15 pM in both “calf” and “normal” interstitia), would have been q_V,-Ctrl_ = (q_TotalVEGF,Normal_, q_TotalVEGF,Calf_) = (0.264, 0.154) molecules/MD/s.

**Figure 3:**
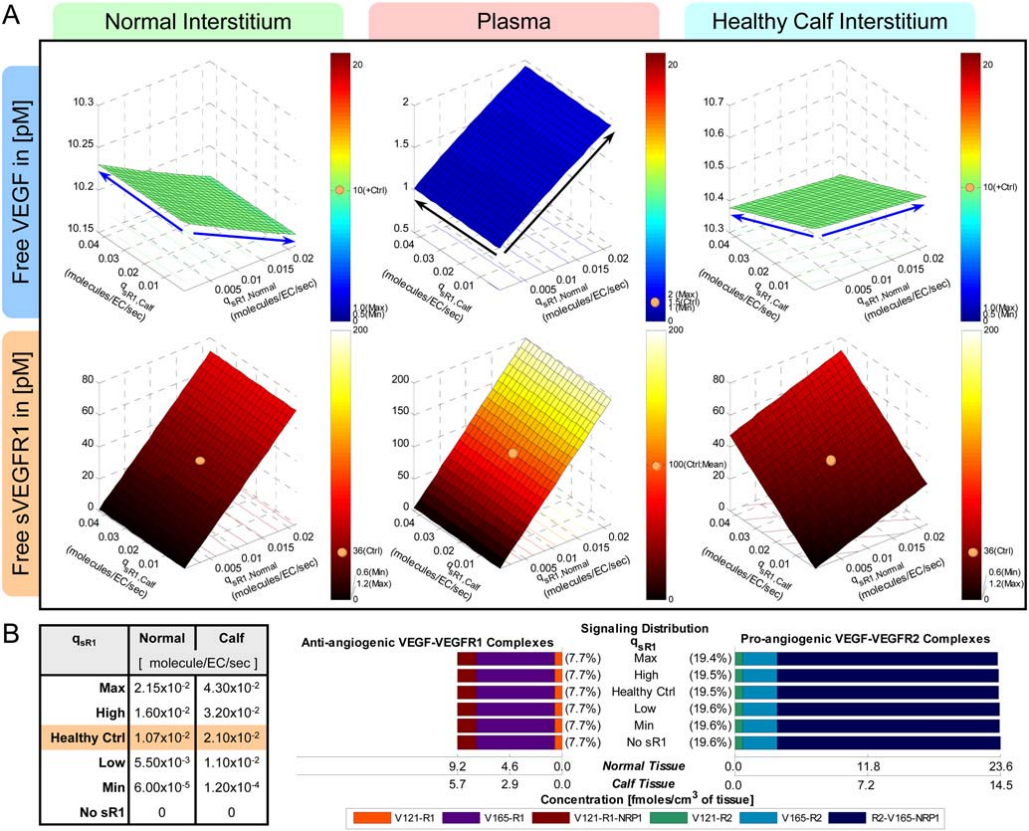
Targeting Control sVEGFR1-Secretion Rates (q_sR1_) for Basal Profile of Healthy Subject. A: *Steady-state sensitivity of plasma and interstitial concentrations of free VEGF (top row) and free sVEGFR1 (bottom row) to q_sR1_ from normal tissue (y-axis) and calf (x-axis)*. *Top row:* Interstitial free VEGF decreased while plasma free VEGF increased with increasing q_sR1_. Blue/black arrows indicate inverse/direct relation between VEGF concentrations and sVEGFR1-secretion rates. *Bottom Row:* The targeted 100 pM of free sVEGFR1 was reached in plasma at the control secretion rates of q_sR1,Ctrl_ = (q_sR1,Normal_, q_sR1,Calf_) = (0.0107,0.0210) molecule/EC/s. Beige spheres mark the interstitial and plasma sVEGFR1 levels reached at q_sR1,Ctrl_. B: *Density of VEGF-VEGFR complexes were insensitive to q_sR1_*. Bracketed percentages are VEGF-bound fractional occupancies of total VEGFR, averaged (range<0.4%) between normal and calf compartments. In figure: ‘+’ = control; ‘max’, ‘min’ and ‘mean’ indicate targeted ranges; ‘EC’ = endothelial cell; ‘V121’ = VEGF_121_; ‘V165’ = VEGF_165_; ‘R1’ = VEGFR1; ‘sR1’ = sVEGFR1; ‘R2’ = VEGFR2.

However, the incorporation of sVEGFR1 expression into the system (Fig. 2A: ‘+sR1’) was found to shift the VEGF profile upwards in the plasma – i.e., if VEGF-secretion rates were fixed at q_V,-Ctrl_, [V]*_pl_* became 2.1 pM once sVEGFR1-secretion rates were high enough to attain a target [sR1]*_pl_* of 100 pM. Hence in seeking our control sVEGFR1-secretion rates, we redefined the control VEGF-secretion rates to q_V,+Ctrl_ = (q_TotalVEGF,Normal_, q_TotalVEGF,Calf_) = (0.1925, 0.1155) molecule/MD/s = (9.3, 5.7)·10^−18^ mole/(cm^3^ tissue)/s, so that [V]*_pl_* would start off at the lower bound of the target range (1 pM) in the absence of sVEGFR1. The steady-state plasma and interstitial free sVEGFR1 concentrations ([sR1]*_pl_*, [sR1]*_IS_*) were then plotted over a range of sVEGFR1-secretion rates from the normal tissue and calf compartments (q_sR1,Normal_ and q_sR1,Calf_) in Fig. 3A, while VEGF-secretion was fixed at q_V,+Ctrl_. The control sVEGFR1-secretion rates that could attain a [sR1]*_pl_* of 100 pM, as well as symmetric [sR1]_IS_ (i.e, 36 pM in both “calf” and “normal” interstitia), were determined to be q_sR1,Ctrl_ = (q_sR1, Normal_, q_sR1,Calf_) = (0.0107,0.0210) molecules/EC/s = (1.3, 1.5)·10^−19^ moles/(cm^3^ tissue)/s. At this q_sR1,Ctrl_, [V]*_pl_* became exactly the targeted 1.5 pM, with [V]*_IS_* now at 10 pM (Fig. 2A: middle row). Although the control secretion rates needed to reach a target set of [V]*_pl_* and [sR1]*_pl_* were not unique – i.e., higher sVEGFR1-secretion rates could theoretically also have elevated [V]*_pl_* from below 1 pM in absence of sVEGFR1 to 1.5 pM in presence of sVEGFR1 – we settled on this set of q_V,+Ctrl_ and q_sR1,Ctrl_ (Table 11) such that [V]*_pl_* remained within experimental range, with or without sVEGFR1 expression in the simulation.

**Table 11:** VEGF- and sVEGFR1-Secretion Rates (Fitted Kinetic Parameters).

	Normal	Healthy Calf	Units
**Secretion of VEGF from PCs into IS**			
q_V121_∶q_V165_	1∶10	1∶10	
q_V121_	0.0175	0.0105	molecule/MD/s
	8.44·10^−19^	5.21·10^−19^	mole/(cm^3^ tissue)/s
q_V165_	0.175	0.105	molecule/MD/s
	8.44·10^−18^	2.73·10^−18^	mole/(cm^3^ tissue)/s
**Secretion of sVEGFR1 from ECs into IS**			
q_sR1_	0.0107	0.021	molecule/EC/s
	1.30·10^−19^	1.53·10^−19^	mole/(cm^3^ tissue)/s

MD = myonuclear domain (parenchymal cell unit).

EC = endothelial cells.

IS = interstitial space.

Conversions are based on geometry parameters from Table 1:
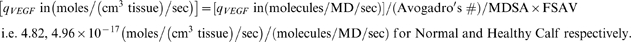


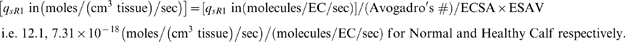

### 1.2. Basal distribution profiles

Using the aforementioned control secretion rates, the following molecular distributions and occupancy profiles were achieved at steady state. (See “Healthy Ctrl” in Supplemental Fig. S1 for details.)

#### i. VEGF distribution

The ratio of free VEGF_121_∶VEGF_165_ in plasma and interstitial concentrations were 1∶10.8 and 1∶10.6 respectively (Fig. S1-A), just slightly lower than the original isoform ratio of the VEGF-secretion rate (1∶10). In the blood, both VEGF_121_ and VEGF_165_ were 23% free and 77% bound to sVEGFR1 (Fig. S1-C,D). This predicted complexed fraction of VEGF was much higher than that experimentally measured by Belgore *et al.* in healthy human plasma (∼4% mole fraction, based on 113 pg/mL free VEGF and 18 pg/mL complexed sVEGFR1) [45]. In the normal interstitium, VEGF_121_∶VEGF_165_ were 0.9%∶0.6% free, 0.8%∶0.6% sVEGFR1-bound, 98.3%∶63.0% surface receptor-bound, and 0%∶35.8% matrix-bound (Fig. S1-C,D). In the calf interstitium, VEGF_121_∶VEGF_165_ were 4.8%∶1.4% free, 5.0%∶1.5% sVEGFR1-bound, 90.2%∶24.7% surface receptor-bound, and 0%∶72.4% matrix-bound (Fig. S1-C,D). In other words, while VEGF was mostly bound to sVEGFR1 in blood, almost all extracellular VEGF in muscle tissue was surface receptor- or matrix-bound. Compared to our previous single-compartment and multi-compartment results in the absence of sVEGFR1 [54], [59], the addition of sVEGFR1 into the VEGF system did not significantly alter the predicted total VEGF distribution in normal muscle tissue.

#### ii. sVEGFR1 distribution

In the blood, 95% of total sVEGFR1 were free, with only 5% complexed to VEGF (Fig. S1-B). This predicted VEGF-occupancy of total plasma sVEGFR1 was again higher than that experimentally measured in healthy human plasma (∼0.65% mole fraction, based on 21 ng/mL free sVEGFR1 vs. 18 pg/mL complexed sVEGFR1) [45]. In the normal∶calf interstitium, sVEGFR1 were 1.6%∶1.9% free, 97.4% matrix-bound, 0.4%∶0.6% VEGF-bound (with or without NRP1 coupling), and 0.6%∶0.1% NRP1-bound.

#### iii. Inter-compartmental distributions

Due to geometrical differences between histological cross-sections of vastus lateralis and gastrocnemius, our normal muscle was characterized with higher total receptor density (due to 11.3% higher myocyte volume fraction, Table 1) than that in calf muscle, while our calf muscle was characterized with higher total matrix site density (due to 8% larger available interstitial fluid volume fraction, Table 1) than that in normal muscle. Therefore the normal compartment had higher amount of total extracellular VEGF_121_ (mostly surface receptor-bound), but lower amounts of total extracellular VEGF_165_ and sVEGFR1 (mostly matrix-bound), per tissue volume than the calf compartment (Fig. S1-B,C,D).

#### iv. Occupancy of matrix sites

The fractional occupancies of total matrix sites were uniformly minute across ECM, EBM and PBM, as well as between normal and calf compartments (differences <0.001%) – only 0.04% VEGF_165_-occupied and 0.15% sVEGFR1-occupied – leaving 99.81% unoccupied (Fig. S1-E,F,G). These fractional occupancies remained characteristically low (<0.6%) throughout sensitivity analyses.

#### v. Occupancy of VEGFRs and NRP1

The fractional occupancies of total surface receptors were also consistent between normal and calf compartments (differences <1%): (i) total VEGFR1 were 26% free, 66% NRP1-coupled but not VEGF-ligated, and 8% VEGF-ligated (Fig. S1-H); (ii) total VEGFR2 were 80% free and 20% VEGF-ligated (Fig. S1-I); (iii) total NRP1 were 16% free, 0.3% sVEGFR1-bound with or without VEGF_121_, 0.1% VEGF_165_-bound, 66% coupled to unactivated VEGFR1 (i.e., VEGFR1-NRP1), and 18% coupled to signaling VEGFRs (i.e., VEGF_121_-VEGFR1-NRP1 or VEGFR2-VEGF_165_-NRP1) (Fig. S1-J).

### 1.3. Physiological variation in calf volume-of-interest

Within a threefold increase in volume of the “healthy calf muscle” compartment (up to 2,604 cm^3^), along with corresponding decreases in the “normal compartment” volume (down to 58,717 cm^3^), all control predictions of VEGF and sVEGFR1 distributions remained consistent (to within 1%), while receptor occupancy profiles remain unchanged. This implied that in cases that require modeling of bigger calf regions-of-interest, such as to include the tibialis anterior, or to study bilateral calves, the geometric differences between our calf vs. normal muscle parameterizations would not cause deviations in the overall baseline healthy profile attained by the stated set of control secretion rates.

### 1.4. Basal concentration gradients & flow profiles

**Figure 4:**
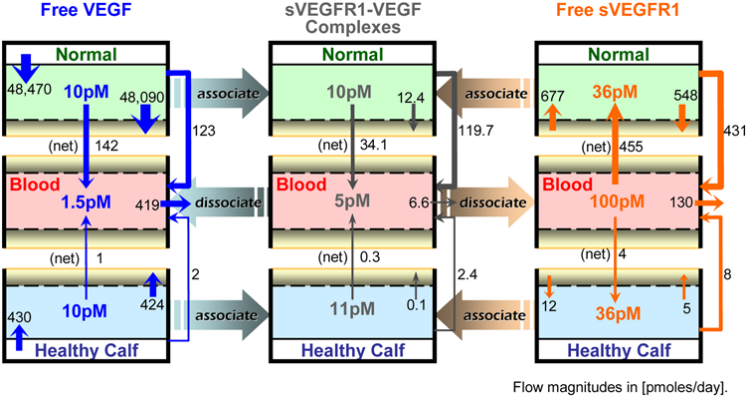
Basal Steady-State Flow Profiles of Free VEGF (left), sVEGFR1-VEGF Complexes (middle), Free sVEGFR1 (right). *Solid-colored arrows* represent intra-compartmental flows (i.e., secretion, internalization) and inter-compartmental flows (i.e., net vascular permeability, lymph flow, plasma clearance), with their relative magnitudes reflected by arrow widths. *Color-graded arrows* between columns indicate mass transfer flows between species (i.e., net association of free VEGF and free sVEGFR1 to form sVEGFR1-VEGF complexes, or net dissociation of the complex back into its constituents).

Fig. 4 summarizes the inter- and intra-compartmental flow balance of soluble species at basal secretion rates; as will be shown in subsequent sections, the net directions and relative magnitudes of these mass flows dictated how the system responded to parameter perturbations. A key difference was noted between the VEGF and sVEGFR1 flows at steady state: most interstitial VEGF was internalized locally, via complex formation with abluminal VEGFRs, before it had a chance to permeate into the plasma, contributing to its lower plasma concentrations; whereas interstitial sVEGFR1, apart from its abluminal internalization, had an equally significant route of escape via lymph flow which contributed to its higher plasma concentration. Consequently, the transendothelial (plasma vs. interstitial) concentration gradients were differentially established, such that free VEGF and sVEGFR1-VEGF complexes experienced net intravastion (IS to plasma) at control, while free sVEGFR1 had an overall tendency to extravasate (plasma to IS) at control. Integral to the flow balances were the net mass transfers between the three soluble species: interstitially, the steady-state inflows for both free VEGF and sVEGFR1 outweighed their respective outflows, signifying a net tendency for them to associate and form sVEGFR1-VEGF complexes; in the plasma, the steady-state inflows for VEGF and sVEGFR1 were less than their respective outflows, indicative of an additional source from net dissociation of sVEGFR1-VEGF complexes.

### 2. Increasing VEGF- or sVEGFR1-secretion rates did not systemically lower free sVEGFR1 or free VEGF concentrations respectively

### 2.1. System sensitivity to VEGF-secretion rates

First we examined the VEGF response to increasing VEGF-secretion rates as illustrated in Fig. 2A. [V]*_IS,Normal_* and [V]*_IS,Calf_* were essentially determined by their respective local VEGF secretion rates, q_TotalVEGF,Normal_ and q_TotalVEGF,Calf_. This was expected with [V]*_IS_*, since the intracompartmental flows (secretion and internalization) of VEGF dominated over its intercompartmental flows (vascular permeability and lymphatic drainage) in magnitude (Fig. 4). Thus [V]*_IS_* was greatly sensitive to local changes in q_TotalVEGF_, but relatively insensitive to q_TotalVEGF_ of the other compartment due to weak intercompartmental communication. Similarly, [V]*_pl_* was increasingly dependent on q_TotalVEGF,Normal_ but largely insensitive to q_TotalVEGF,Calf_, following the much larger intravasation flow from the normal compartment relative to that from the calf (Fig. 4).

On the other hand, sVEGFR1 concentrations were affected by VEGF-secretion rates in two asymmetrical ways. Firstly, the response to increasing q_TotalVEGF,Calf_ was expected from previous flow analysis (Fig. 4): a surge in [V]*_IS,Calf_* was compensated by increased complex formation, lowering [sR1]*_IS,Calf_* in the process of association (Fig. 2A). Subsequent intravasation of sVEGFR1-VEGF complexes and their dissociation in plasma was too modest to affect [sR1]*_pl_* or [sR1]*_IS,Normal_*. A second mode of response occurred with increasing q_TotalVEGF,Normal_, beginning with an elevated [V]*_IS,Normal_* which promoted VEGFR2-VEGF_165_-NRP1 formation, thereby diminishing the availability of free NRP1 to bind free sVEGFR1. As a result of decreased sVEGFR1-internalization via NRP1-complexes, interstitial free sVEGFR1 actually increased globally (in both [sR1]*_IS,Normal_* and [sR1]*_IS,Calf_*) via [sR1]*_pl_* (Fig. 2A). The asymmetry was a consequence of the calf compartment having less endothelial surface area per volume and thus lower total surface receptor densities than the normal muscle compartment (supplemental Fig. S1), such that the second mechanism involving NRP1-VEGFR2 coupling was not sizeable enough to override the first mechanism involving sVEGFR1-VEGF association.

Lastly, the density of VEGF-ligated signaling complexes on the abluminal surface of the endothelium positively correlated with [V]*_IS_*. Consequently, increasing q_TotalVEGF_ intensified both VEGFR1 and VEGFR2 signaling profiles (Fig. 2B).

### 2.2. System sensitivity to sVEGFR1-secretion rates

Fig. 3A illustrates the VEGF and sVEGFR1 responses to increasing sVEGFR1-secretion rates. [sR1]*_IS,Normal_* and [sR1]*_IS,Calf_* were predicted to most significantly depend on their local sVEGFR1-secretion rates, q_sR1,Normal_ and q_sR1,Calf_ respectively, in a linear fashion. Flow analysis (Fig. 4) suggested that the elevated interstitial free sVEGFR1 would then associate with free VEGF to form sVEGFR1-VEGF complexes, which accounted for the slight decreases in [V]*_IS_* in the direction of increasing local q_sR1_. Furthermore, the complexes formed in the interstitium were expected to intravasate and dissociate in the plasma, as confirmed by the rise in [V]*_pl_* and [sR1]*_pl_* in the direction of increasing q_sR1,Normal_. The cycle completes with part of the elevated [sR1]*_pl_* extravasating back into the interstitium, hence accounting for the increase in [sR1]*_IS,Calf_* in the direction of increasing q_sR1,Normal_. Thus unlike VEGF in the previous subsection, interstitial sVEGFR1 (e.g., [sR1]*_IS,Calf_*) was able to respond to distal changes (e.g., q_sR1,Normal_). The asymmetry where increasing q_sR1,Calf_ did not elevate [sR1]*_IS,Normal_* was due to the fact that the intravasation flow of the complex from the calf was insufficient to elevate [sR1]*_pl_* on its own. Finally, the signaling profiles did not change significantly as a function of q_sR1_ (Fig 3B), reflective of the minute changes in [V]*_IS_* already described.

### 3. Receptor densities and ratios affected plasma and interstitial concentrations of VEGF and sVEGFR1, as well as surface-bound VEGFR occupancy

**Figure 5:**
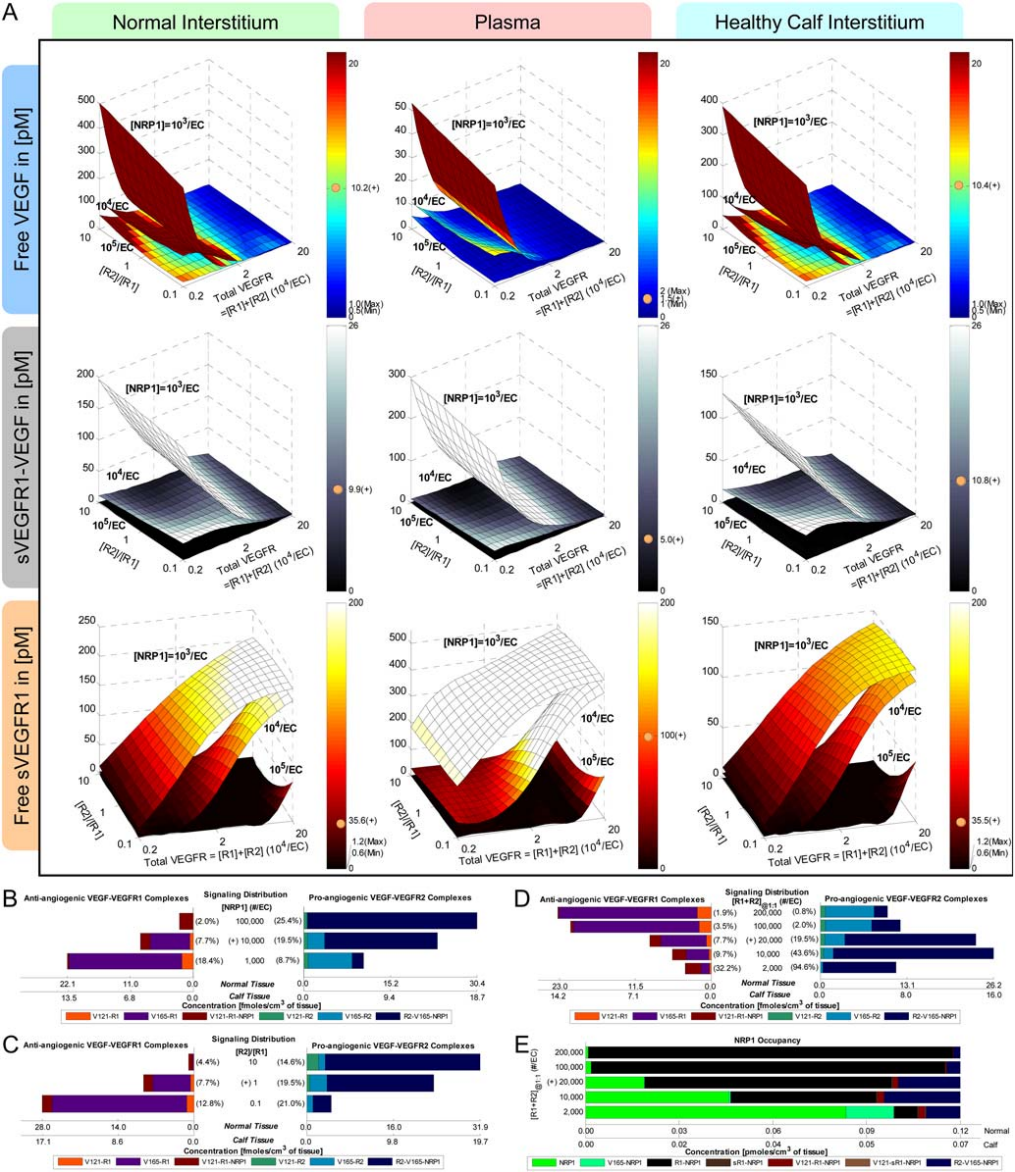
Steady-State Sensitivity to Receptor Density. A. Sensitivity of plasma and interstitial concentrations of free VEGF, free sR1, and sR1-VEGF complex to NRP1 density (3 surfaces), VEGFR1∶VEGFR2 density ratio (x-axis), and total VEGFR density (y-axis). B–D. Sensitivity of signaling complex distribution to NRP1 density (B), ratio of VEGFR2∶VEGFR1 density ratio (C), and total VEGFR density (D). Bracketed percentages are VEGF-bound fractional occupancies of total VEGFR1 (left) and total VEGFR2 (right), averaged (range <0.9%) between normal and calf compartments. E. Sensitivity of NRP1 distribution to total VEGFR density. ‘+’ = control; ‘max’ and ‘min’ bound targeted ranges; ‘EC’ = endothelial cell; ‘V121’ = VEGF_121_; ‘V165’ = VEGF_165_; ‘R1’ = VEGFR1; ‘sR1’ = sVEGFR1; ‘R2’ = VEGFR2.

Receptor densities and ratios were varied over two orders of magnitude about the healthy control values, while keeping VEGF- and sVEGFR1-secretion rates fixed, in a steady-state sensitivity analysis to predict system response to physiological/pathological regulation of receptor expression levels. Interstitial and plasma concentrations of free VEGF and free sVEGFR1 were found to be sensitive over most of the tested ranges (Fig. 5A), as described in further mechanistic detail below. Overall, increasing NRP1 density, increasing VEGFR2∶VEGFR1 ratio (denoted as [R2]/[R1]), and decreasing total VEGFR density all steepened plasma vs. interstitium gradients (i.e., farther from 1∶1) for VEGF concentrations; while increases in all three parameters reduced sVEGFR1 gradients (i.e., closer to 1∶1). In addition, higher NRP1 density or [R2]/[R1] favored a net shift towards pro-angiogenic signaling; while total VEGFR densities within the same order of magnitude as NRP1 density were predicted to be optimal for overall pro-angiogenic signaling (Fig. 5B–D).

### 3.1. Sensitivity to neuropilin-1 density

Higher NRP1 densities were generally associated with lower concentrations of all soluble species, since NRP1 was a vehicle for internalizing VEGF and sVEGFR1 via endothelial VEGF_121_-NRP1, VEGF_165_-NRP1, sVEGFR1-NRP1, VEGF_121_-VEGFR1-NRP1 complexes. For free VEGF, this was most evident at low total VEGFR density (Fig. 5A), when the alternative internalization route for VEGF via VEGFR was minimized. For free sVEGFR1, this was most evident at high total VEGFR density (Fig. 5A), when free VEGF remained low to reduce the secondary effects of sVEGFR1-VEGF complex formation. Secondarily, declines in sVEGFR1-VEGF complex concentrations followed increases in NRP1 density (Fig. 5A), as NRP1 competed with sVEGFR1 for binding with free VEGF.

Moreover, increasing NRP1 density shifted the VEGF-signaling profiles as shown in Fig. 5B. The overall drop in “anti-angiogenic potential”, as represented by ligated VEGFR1 complexes, can be explained by NRP1's high affinity for VEGFR1. At 10^5^ NRP1/EC, almost all (97%) VEGFR1 became part of unligated VEGFR1-NRP1 complexes – a shift that dramatically reduced the availability of VEGFR1 for VEGF_165_-ligation (Fig. 5B). On the other hand, the overall rise in “pro-angiogenic potential”, as represented by ligated VEGFR2 complexes, can be explained by NRP1's role as a co-receptor in presenting NRP1-bound VEGF_165_ to VEGFR2, as well as in stabilizing VEGF_165_-VEGFR2 through their triplet configuration. All together, these synergistic functions of NRP1 in reducing anti-angiogenic complexes and promoting pro-angiogenic complexes, were in tune with computational predictions from our previous studies in the absence of sVEGFR1 [54].

### 3.2. Sensitivity to VEGFR2∶VEGFR1 density ratio ([R2]/[R1])

Although higher NRP1 density in general lowered free VEGF concentrations predominantly through enhanced internalization of VEGF-bound NRP1, exceptions were noted in the region of low [R2]/[R1] (<1), roughly between 10,000 to 20,000 total VEGFR/EC, where free VEGF concentrations were apparently higher at 10,000 NRP1/EC than at 1,000 NRP1/EC (Fig. 5A). In this region, the greater abundance of VEGFR1 gave more prominence to NRP1's tendency to competitively shift the distribution of total VEGFR1 towards formation of unligated VEGFR1-NRP1 complexes, in the process freeing VEGF that had been bound to uncoupled VEGFR1, hence elevating free VEGF concentrations.

For the independent increase of [R2]/[R1] while fixing total receptors at control densities, the following concentration changes were observed in all three fluid compartments (Fig. 5A), originating from the tissues' interstitial fluids and propagated into the plasma: (i) free VEGF_121_ increased – due to a net decrease in internalization force (reduction in VEGF_121_-VEGFR1 and VEGF_121_-VEGFR1-NRP1 outweighed increase in VEGF_121_-VEGFR2) (Fig. 5C); (ii) free VEGF_165_ decreased – due to a net increase in internalization force (increase in VEGF_165_-VEGFR2 and VEGFR2-VEGF_165_-NRP1 overshadowed reduction in VEGF_165_-VEGFR1) (Fig. 5C); (iii) an overall decrease in free total VEGF – despite an increasing fraction of isoform 121 (up to VEGF_121_∶VEGF_165_ = 1∶3 at [R2]/[R1] = 10); and (iv) free sVEGFR1 decreased – due to dissociation of NRP1 from VEGFR1-NRP1 complexes to become available for sVEGFR1-binding and internalization (data not shown).

### 3.3. Sensitivity to total VEGFR density ([R1]+[R2])

Our simulations presented several theoretical indications that the VEGF/sVEGFR1 system has optimal operating range around the VEGFR1∶VEGFR2∶NRP1 receptor density ratio of 1∶1∶1. Firstly, in examining the free sVEGFR1 concentration surfaces shown in Fig. 5A, the region of maximal or linear gain for each surface always spanned total VEGFR densities near the same order of magnitude as NRP1 density. In fact, the sVEGFR1 concentration surfaces for 10,000 NRP1/EC were not only sigmoidal over the examined total VEGFR density range, centered about [R1]+[R2] = 20,000/EC, they were also sigmoidal in the direction of VEGFR ratio, centered about 1∶1. Similarly, of the free VEGF concentration surfaces, the sigmoidal surface for 10,000 NRP/EC had the closest operating range over [R1]+[R2] = 20,000/EC. A second indication was based on the observation that the formation of signaling VEGFR2 complexes was biphasic in the direction of total VEGFR density, allowing the most efficient net “pro-angiogenic potential” to be reached within the order of 10,000 total VEGFR/EC (Fig. 5D).

Mechanistically, the dependence of signaling profiles on total VEGFR density as depicted in Fig. 5D could be explained by two forces: (1) the direct effects of the sheer increase in number of VEGFRs available for VEGF ligation; (2) the indirect effects of VEGFR's density ratio relative to NRP1 which was fixed at 10,000/EC. The former effects increased quantities of non-NRP1-coupled VEGF-VEGFR complexes with increasing total VEGFR density, despite the diminishing fractional occupancies of VEGFRs (Fig. 5D). The latter effects stemmed from an increasing proportion of total NRP1 being used up in formation of unligated VEGFR1-NRP1 complexes with increasing total VEGFR density (Fig. 5E). This meant that the higher the total VEGFR density, the less free NRP1 were available to form VEGFR2-VEGF_165_-NRP1 and VEGF_121_-VEGFR1-NRP1, hence the generally decreasing contribution of these species in their respective signaling profiles (Fig. 5D). The opposing trend in the biphasic nature of VEGFR2-VEGF_165_-NRP1 (Fig. 5D) came from the fact that at very low total VEGFR density, quantities of VEGFR2 were so limited that there was an unusual population of VEGF_165_-NRP1 left at steady state for 2,000 VEGFR/EC (Fig. 5E) with no VEGFR2 to present to. Thus explained the quick jump in VEGFR2-VEGF_165_-NRP1 as soon as VEGFR2 were available at 10,000 VEGFR/EC (Fig. 5D,E).

### 4. VEGFRs' affinities for VEGF affected free sVEGFR1 concentrations via NRP1 availability; NRP1's affinity for VEGF_121_ was inconsequential

This section explores system sensitivity to the effective (microenvironment-dependent) dissociation constants of VEGF from its receptors over the wide ranges reported in literature.

### 4.1. Sensitivity to VEGF-binding affinity of VEGFR1 and VEGFR2

**Figure 6:**
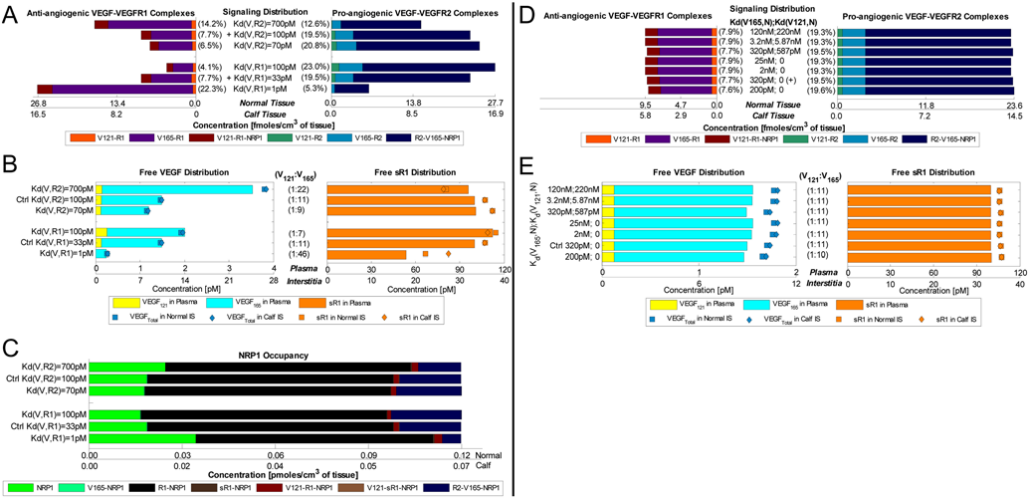
Steady-State Sensitivity to VEGF-Binding Affinities of Cell Surface Receptors: VEGFR1, VEGFR2 and NRP1. A. Higher VEGF-VEGFR1 affinity and VEGF-VEGFR2 affinity respectively shifted the signaling profile towards anti- and pro-angiogenic complexes. B. Free VEGF levels lowered with increasing VEGF-binding affinity of either VEGFR1 or VEGFR2; while free sVEGFR1 levels rose and fell with increasing VEGF-VEGFR1 and VEGF-VEGFR2 affinity respectively. C. Availability of unbound NRP1 changed inversely with the amount of VEGFR2-VEGF_165_-NRP1 formed. Free VEGF and sVEGFR1 levels (D), as well as signaling profiles (E), were largely insensitive to NRP1's direct binding-affinity with VEGF. Bracketed percentages in A and D are VEGF-bound fractional occupancies of total VEGFR, averaged (range <0.3%) between normal and calf compartments. ‘+’/‘Ctrl’ = control; ‘K_d_(V,R)’ = dissociation constant between VEGF and VEGFR; ‘K_d_(V121/V165,N)’ = dissociation constants between VEGF_121_/VEGF_165_ and NRP1; ‘R1’ = VEGFR1; ‘sR1’ = sVEGFR1; ‘R2’ = VEGFR2.

The shifts in signaling profiles were as expected from the competitive binding of VEGF between VEGFRs: increasing one VEGFR's VEGF-affinity boosted formation of its signaling complexes to the detriment of the other VEGFR's complex formation (Fig. 6A). As for the soluble species, total free VEGF in all compartments decreased with increasing VEGF-binding affinity of either VEGFR1 or VEGFR2 – presumably through enhanced internalization of complexed VEGF (Fig. 6B). Free sVEGFR1 concentrations, however, changed in opposite directions: lowered with increasing VEGF-VEGFR1 affinity but rose with increasing VEGF-VEGFR2 affinity. The directional change in free sVEGFR1 thus followed that of VEGFR2-VEGF_165_-NRP1 complexes (Fig. 6A) – i.e., the more VEGFR2-VEGF_165_-NRP1 complexes formed, the less unbound NRP1 available for binding and internalization of free sVEGFR1 (Fig. 6C).

### 4.2. Sensitivity to VEGF-binding affinity of NRP1

Lowering NRP1's affinity for VEGF_165_ over two orders of magnitude – K_d_(V_165_,N) from 200 pM to 25 nM –caused only opposing changes of up to 0.3% in the VEGF-bound fractional occupancies of VEGFR1 and VEGFR2 (Fig. 6D). The minute attenuation of pro-angiogenic potential reflected a declining availability of VEGF_165_-bound NRP1s for coupling with VEGFR2, hence the diminishing quantities of VEGFR2-VEGF_165_-NRP1 (Fig. 6D). Simultaneously in all fluid volumes, free VEGF_121_ levels remained consistent while free VEGF_165_, presumably released from NRP1s, elevated slightly (plasma data shown in Fig. 6E). This in turn increased availability of free VEGF_165_ for direct VEGFR1-binding (Fig. 6D).

Our simulations also predicted inconsequential effects from incorporating the newly purported binding interaction between NRP1 and VEGF_121_. Specifically, when modeling both VEGF_121_- and VEGF_165_-affinities of NRP1 at the low affinities cited by Pan *et al.* (220 nM and 120 nM respectively [105]), there were no remarkable changes in signaling profiles (Fig. 6D) nor concentrations of free soluble species (Fig. 6E) compared to simulation results with no VEGF_121_-NRP1 binding but weak VEGF_165_-NRP1 binding. Furthermore, when keeping VEGF_165_-NRP1 affinity at control (320 pM) and introducing VEGF_121_-NRP1 binding at an affinity 1.83× higher than that (in accordance with the ratio reported by Pan *et al.*), all system distributions were almost identical to those at control – because even then, the steady-state population of VEGF_121_-NRP1 only represented an insignificant 0.7% of total VEGF_121_ in muscle tissues.

Thus, as long as VEGF had a lower affinity to NRP1 than to VEGFRs, the system was largely insensitive to variations in the VEGF-binding affinities of NRP1. This suggested that mechanistically, the significance of NRP1 as a co-receptor in the VEGF system could be largely attributed to NRP1's strength in coupling VEGFRs rather than its direct affinity for VEGF.

### 5. Densities and VEGF-affinity of interstitial matrix sites affected only matrix-bound reservoirs of VEGF_165_ and sVEGFR1

**Figure 7:**
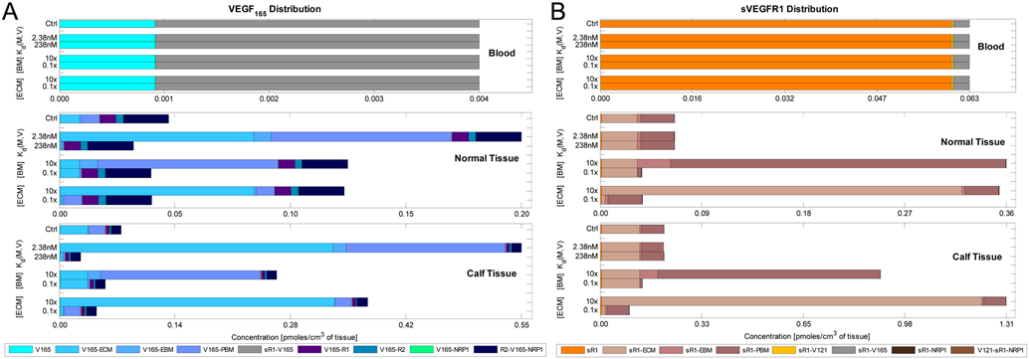
Sensitivity to Density & VEGF-Binding Affinity of Interstitial Matrix Sites for VEGF_165_ & sVEGFR1. Steady-state sensitivity of VEGF_165_ (A) and sVEGFR1 (B) distributions in blood (top), normal tissue (middle) and calf tissue (bottom) compartments to the VEGF_165_-binding affinity (K_d_(M,V)) and densities ([ECM]; [BM]) of interstitial matrix sites. At control: K_d_(M,V) = 23.8 nM; [ECM] = 20 µM; [BM] = 0.75 µM. Concentrations of soluble species (e.g., free VEGF_165_, free sVEGFR1, sVEGFR1-VEGF_165_) and surface VEGFR occupancies (e.g., VEGF_165_-VEGFR1, VEGF_165_-VEGFR2, VEGFR2-VEGF_165_-NRP1) were completely insensitive; whereas the sizes of matrix-bound reservoirs of VEGF_165_ and sVEGFR1 (e.g., VEGF_165_-ECM, sVEGFR1-PBM) were greatly affected. ‘K_d_(M,V)’ = dissociation constant between interstitial matrix sites and VEGF_165_; ‘[ECM]’ and ‘[BM]’ = densities of binding sites for VEGF or sVEGFR1 in extracellular matrix and basement membranes respectively’; ‘Ctrl’ = control; ‘PBM’ = parenchymal BM; ‘EBM’ = endothelial BM; V165 = VEGF_165_; ‘R1’ = VEGFR1; ‘sR1’ = sVEGFR1; ‘R2’ = VEGFR2.

Steady-state analyses showed that fluctuations in the VEGF-binding affinity and densities of interstitial matrix sites had no detectable effects on the concentrations of all soluble species (in both plasma and interstitial fluid), nor on surface receptor occupancies (Fig. 7). In contrast, the quantities of matrix-bound VEGF_165_ and sVEGFR1 increased drastically with increasing matrix site densities (Fig. 7A,B); while the matrix-bound reservoir of VEGF_165_ also grew drastically with higher VEGF_165_-affinity of matrix sites (Fig. 7A). Whereas these effects culminated into several-fold changes in total VEGF_165_ and sVEGFR1 in muscle tissues, the fractional occupancy of total matrix sites remained very low (at a consistent 0.19% irrespective of matrix site densities) and changing minutely with varying VEGF_165_-affinity (up to 0.55% at 10× control *K_d_*(M,V_165_).

### 6. Transport parameters affected concentrations of plasma VEGF, plasma sVEGFR1 and interstitial sVEGFR1, but not surface-bound VEGFR occupancy

**Figure 8:**
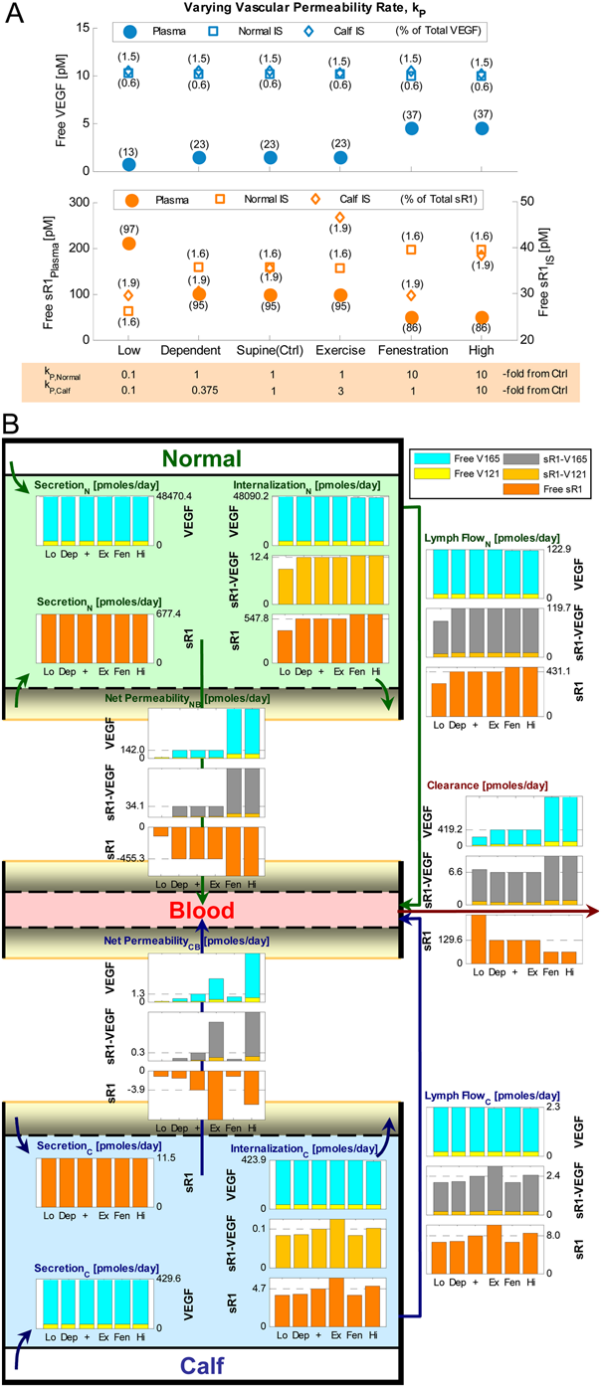
Steady-State Effects of Permeability Rate (*k_P_*) on VEGF and sVEGFR1 Concentrations (A) & Flows (B). In general, with increasing *k_P_*, concentrations changed in the directions that reduced transendothelial gradients: (i) plasma VEGF concentration increased; (ii) plasma sVEGFR1 decreased and interstitial sVEGFR1 increased. Exceptions were observed with localized changes in *k_P_* – e.g., increasing only *k_P,Normal_* from ‘supine(ctrl)’ to ‘fenestration’ enhanced sVEGFR1 extravasation into the normal compartment in expense of that into the calf, causing non-uniform changes in interstitial sVEGFR1 concentrations (increased locally, decreased distally). ‘Lo’ = low; ‘Dep’ = dependent; ‘+’ = supine (control); ‘Ex’ = exercise; ‘Fen’ = fenestration; ‘H’ = high.

**Figure 9:**
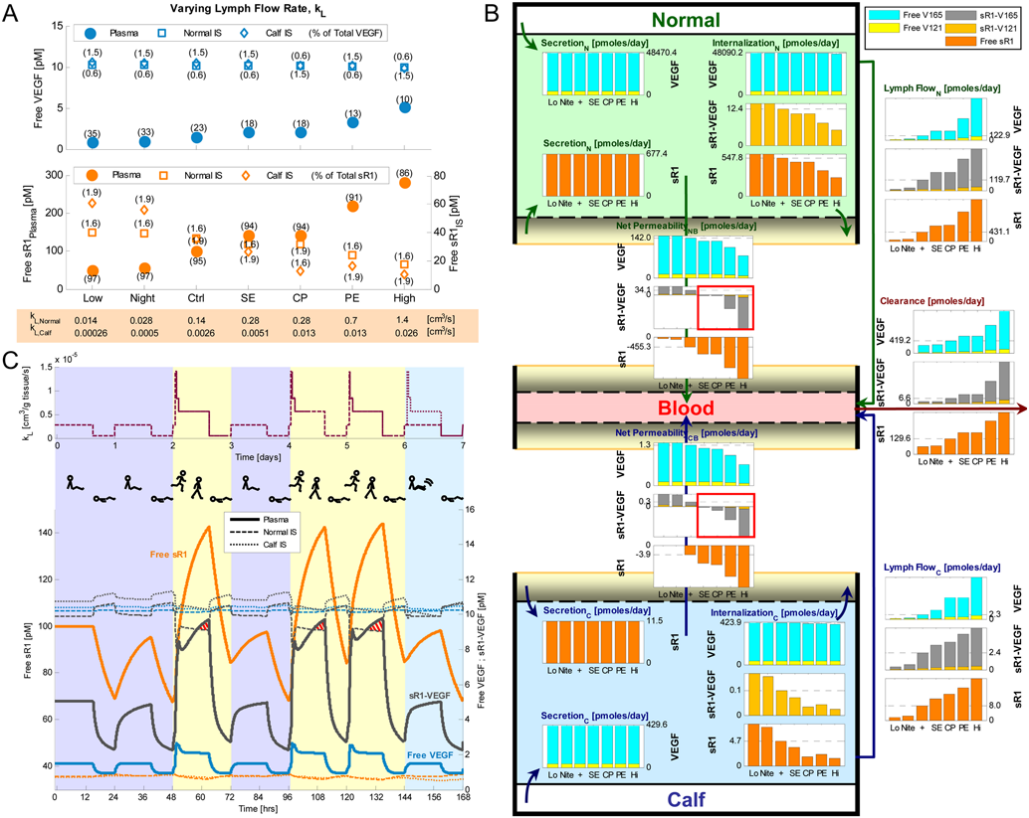
Steady-State and Dynamic Effects of Lymphatic Drainage Rates (*k_L_*). *Steady-state effects of k_L_ on VEGF and sVEGFR1 concentrations* (A) *and flows* (B). Of the three transport parameters (k_P_, k_L_, k_CL_), increasing *k_L_* over two orders of magnitude about the control value resulted in the greatest fluctuations in steady-state concentrations: most significantly elevating plasma VEGF and sVEGFR1, while lowering interstitial sVEGFR1. Exceptions were noted with localized changes in *k_L_* – e.g., with increasing only *k_L,Normal_* from ‘CP’ to ‘PE’, the enhanced flushing of sVEGFR1 from the local interstitium into the plasma eventually spilled over through increased sVEGFR1 extravasation into the opposite compartment to elevate interstitial sVEGFR1 concentration there. In addition, a reversal in permeability flow of sVEGFR1-VEGF complex (red box) was noted for *k_L_* above control. ‘Lo’ = low; ‘Nite’ = night; ‘+’ = supine awake (control); ‘SE’ = whole-body steady exercise; ‘CP’ = calf-only peak exercise; ‘PE’ = whole-body peak exercise; ‘Hi’ = high. *Dynamic Effects of k_L_ on VEGF, sVEGFR1, and sR1-VEGF Concentrations* (C). *k_L_*-driven fluctuations in VEGF and sVEGFR1 attained within physiological diurnal cycles were less than those attained during steady-state analyses but still were of very wide ranges. “Bed-rest days” (purple columns) consisted of 15 hrs of wakefulness limited to supine or sitting postures, followed by 9 hrs of sleep. “Active days” (yellow columns) consisted of 15-hrs of activity starting off with a peak in *k_L_* during early exercise and settling down to a steady running/walking rate, followed by 9 hrs of sleep. Reversed permeability flow of sVEGFR1-VEGF complex (red cross-hatching) was observed in the latter active waking hours. “Calf-limited activity days” (aqua column) are same as “bed-rest days” except that active *k_L_* was induced in the calf during the first 15 hrs of wakefulness. Stick-figure illustrations adapted from Olszewski *et al.* Lymphology 1977, 10(3):178–183.

**Figure 10:**
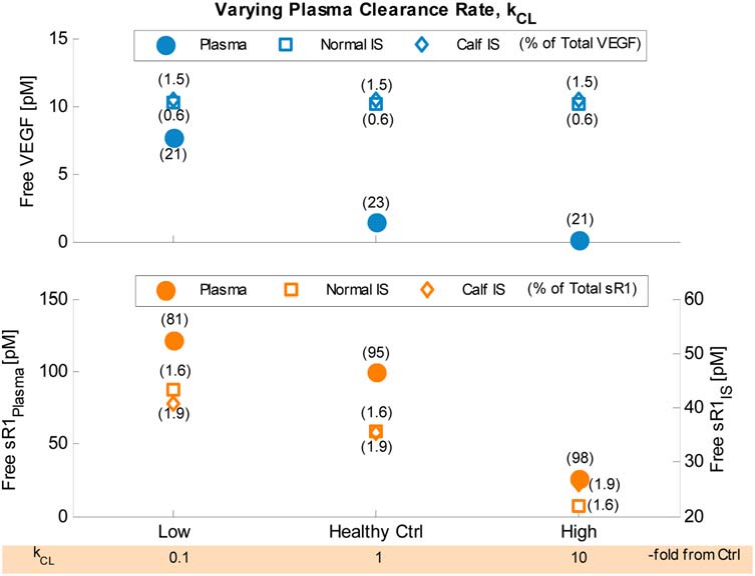
Steady-state Effects of Plasma Clearance Rate (*k_CL_*) on VEGF and sVEGFR1 Concentrations. Increasing *k_CL_* resulted in: (i) drastically lower plasma concentrations of both free VEGF and sVEGFR1, (ii) lower interstitial sVEGFR1 but unchanged interstitial VEGF, and (iii) reduced transendothelial gradient for VEGF but not for sVEGFR1.

Transport parameters were independently varied over two orders of magnitude about control for sensitivity analysis. In plasma, steady-state concentrations of all soluble species were strongly dependent on transport rates, whereas in the interstitium, sVEGFR1 concentration was much more sensitive than VEGF to transport parameters. In general: increasing *k_P_* reduced plasma vs. interstitium gradients (i.e., closer to 1∶1) for both VEGF and sVEGFR1 concentrations (Fig. 8A); increasing *k_L_* lessened VEGF gradients but steepened sVEGFR1 gradients (Fig. 9A); while decreasing *k_CL_* reduced VEGF gradients without much effect on sVEGFR1 gradients (Fig. 10). Additionally, the current model showed that physiological fluctuations in transport parameters were ineffective in altering endothelial VEGFR occupancy, given their minute effect on interstitial free VEGF levels.

### 6.1. Steady-state effects of vascular permeability rates (*k_P_*)

The effect of *k_P_* on free VEGF and sVEGFR1 concentrations (Fig. 8A) could be explained by their associated flow changes (Fig. 8B). Firstly, since the transendothelial VEGF gradient at control favored net intravasation, increasing *k_P_* resulted in significantly higher plasma concentrations of free VEGF. Interstitially, the corresponding decreases in free VEGF were insignificant, as the change in VEGF's transvascular flow was still overshadowed in magnitude by its secretion and internalization flows. Secondly, the transendothelial gradient of sVEGFR1 at control favored net extravasation, hence increasing *k_P_* resulted in: lower plasma concentrations of free sVEGFR1; as well as increased free sVEGFR1 in the interstitium facing the endothelium where *k_P_* was upregulated, at the expense of a decrease in interstitial free sVEGFR1 in the other tissue compartment (e.g., “Control” vs. “Fenestration” in Fig. 8).

VEGF and sVEGFR1 distribution changes were also more drastic in blood than in interstitia. In the blood, the number of complexed sVEGFR1-VEGF increased slightly (e.g., 1.5× from control) with increasing global *k_P_* (e.g., 10× from control). This was sufficient to elevate the fractional occupancy of total sVEGFR1 (e.g., +9%) despite decreasing free sVEGFR1; yet not enough to prevent overall reductions in bound fraction of total VEGF (e.g., -14%) because of the greater increase in free VEGF. In the interstitium, the fractional occupancies of VEGFR1 and VEGFR2 by VEGF decreased <0.5% within the 100-fold increase in *k_P_* tested.

### 6.2. Steady-state effects of lymph flow rates (*k_L_*)

Similarly, *k_L_*-driven changes in steady-state concentrations of free VEGF and sVEGFR1 (Fig. 9A) could be explained by their associated flow changes (Fig. 9B). Since lymph flow represented a unidirectional flushing of VEGF and sVEGFR1 from the interstitium into blood, increasing *k_L_* resulted in higher plasma and lower interstitial concentrations of free VEGF and sVEGFR1. All resulting differences were significant except for interstitial free VEGF, as lymphatic flow was also dwarfed by the secretion and internalization flows in the net balancing of VEGF entering and leaving the interstitia. It was noted that when increasing *k_L_* in one tissue compartment only (e.g., increasing *k_L,N_* only from “Calf Peak Exercise” to “Peak Exercise” in Fig. 9A), the interstitial concentration of free sVEGFR1 only decreased locally (e.g., normal), while slightly increased in the other tissue compartment (e.g., calf). This was due to a secondary “spill-over” of the elevated plasma free sVEGFR1 through increased extravasation into the distal compartment. This may suggest that if increasing *k_L_* were to be explored as a therapeutic means to alleviate calf tissue accumulation of sVEGFR1, local lymphatic flushing as induced by leg exercise may be more productive than whole-body exercise (Fig. 9A).

Distribution changes were again more evident in the blood: the simultaneous elevations in free VEGF and sVEGFR1, due to increasing global *k_L_* (e.g., 10× from control), in turn synergistically increased sVEGFR1-VEGF complex formation (e.g., 88× from control), which elevated the complexed fractions of both VEGF (e.g., +13%) and sVEGFR1 (e.g., +8.7%). In addition, a reversal in permeability flow of sVEGFR1-VEGF complex, from net intravasation to net extravasation, was also observed upon increasing *k_L_* from control to steady exercise rate (Fig. 9B). In the interstitium, the fractional VEGF-occupancies of VEGFR1 and VEGFR2 decreased <1% within the 100-fold increase in *k_L_* tested.

While steady-state analyses showed that plasma concentrations of sVEGFR1 and VEGF could vary up to 164 pM and 2.4 pM respectively (>150% about controls) over the physiological range of *k_L_*, we further examined whether concentration ranges of these magnitudes were attainable within physiological time-course.

### 6.3. Dynamic effects of lymph flow rates (*k_L_*)

A dynamic simulation of the diurnal changes of *k_L_* over a combination of “bed-rest days” and “active days”, as illustrated in Fig. 9C, suggested that physiological variation of *k_L_* over the course of a day can still account for significant variation in plasma concentrations of VEGF and sVEGFR1. In blood, dynamic fluctuations in free sVEGFR1 always eclipsed that of free VEGF in amplitude, at up to 76 vs. 1.65 pM (∼76% vs. 110% about controls). In the interstitia, diurnal ranges of VEGF and sVEGFR1 were much subdued compared to steady-state ranges: free VEGF varied up to 0.2 pM (∼2% about controls), with negligible effects on VEGF-VEGFR complex formation; while free sVEGFR1 varied up to 2.5 pM (∼7% about controls).

It was noted that sVEGFR1 equilibrated slower than VEGF, such that sVEGFR1 never fully re-established its steady state within the day, while VEGF reached its new steady state within hours. This differential characteristic time between VEGF and sVEGFR1 accounts for the unique responses of the three soluble species to activity-dependent changes in *k_L_* during “active days”. From the waking hour (e.g., 48^th^ h in Fig. 9C), plasma concentration of free sVEGFR1 followed a steady rise over the next 15 h before the onset of sleep. On the other hand, plasma free VEGF experienced a much steeper initial rise to a transient peak that closely accompanied the *k_L_* peak of early exercise, but soon falls into a lower plateau as *k_L_* also settled to its own steady state of normal activity. As for the sVEGFR1-VEGF complex in plasma, its steep initial peak and dip followed those of free VEGF, while a latter steady rise was driven by the persistent net influx of free sVEGFR1. In fact, plasma sVEGFR1-VEGF overtook interstitial sVEGFR1-VEGF at some point near the latter active hours of wakefulness, whereupon the permeability flow of the complex reversed in direction to become a net extravasation until the onset of sleep (Fig. 9C).

In summary, a 15-h period of daytime activity could elevate plasma concentrations to 42 pM and 1.1 pM above control for free sVEGFR1 and VEGF respectively. However, 9 h of sleep was adequate for plasma free sVEGFR1 to fall back to below control levels regardless of whether the subject was active or inactive during waking hours (−32 pM after bed-rest vs. −16 pM after activity). Hence, consecutive “active days” negligibly enhanced the peak sVEGFR1 level attainable in subsequent “active days” (+44 pM sVEGFR1; +1.0 pM VEGF relative to control).

### 6.4. Steady-state effects of plasma clearance rates (*k_CL_*)

Increasing direct clearance from blood (*k_CL_*) drastically lowered free VEGF and sVEGFR1 concentrations in plasma (Fig. 10). These primary effects in blood in turn were propagated into the interstitium through permeability, i.e., via reduced net sVEGFR1 extravasation and upregulated net VEGF intravasation. Consequently, interstitial concentrations of free sVEGFR1 were significantly lowered as well; though decreases in interstitial free VEGF were negligible, again because changes in intercompartmental flows were masked by the predominant secretion and internalization flows of VEGF.

Distribution analysis in the blood showed that the number of sVEGFR1-VEGF complexes reduced proportionally to free VEGF (sVEGFR1-complexed fraction of total VEGF was consistent within 2%) while the fractional occupancy of sVEGFR1 dropped from 19% to 2% over the 100-fold variation in *k_CL_*. In the interstitium, the fractional VEGF-occupancies of VEGFRs decreased <0.2% for the *k_CL_* range tested.

## Discussion

### Healthy baseline: transendothelial gradients of VEGF and sVEGFR1

This paper documented our development of an original *in silico* multi-tissue model for simulating the systemic distributions of VEGF and sVEGFR1 for a healthy subject. The unexplained large variability in literature data of measured plasma concentrations of VEGF and sVEGFR1 in healthy subjects necessitates a thorough examination of inter-study differences in experimental protocol. Here in this study, we have chosen to target the plasma concentrations of [V]*_pl_* = 1.5 pM (converted for 46-kDa VEGF homodimers) and [sR1]*_pl_* = 100 pM (converted for 200-kDa sVEGFR1 homodimers). At the control secretion rates of VEGF and sVEGFR1 needed to reproduce these selected plasma concentrations, our predicted interstitial concentrations ([V]*_IS_* = 10 pM; [sR1]*_IS_* = 36 pM) were inconsistent with levels expected from microdialysis data (∼1 pM [135], [136]). Technical concerns have been raised regarding the use of microdialysis membranes of high molecular-weight cut-offs to study macromolecules such as proteins, including compromised spatial resolutions associated with large probe sizes and unwanted ultrafiltration that could alter interstitial space compositions [139]. Until independent experimental validation for interstitial concentrations could be obtained using methods other than microdialysis, we propose several *in silico* assumptions-based sources of discrepancy between our predicted observations vs. microdialysis data for plasma VEGF below. They fall under these categories: (1) interstitium-to-blood transporters of VEGF; (2) blood sources of VEGF or sVEGFR1; (3) interstitial sinks of VEGF and sVEGFR1; and (4) organ subdivisions of body compartment.

First of all, an unexpected consequence of introducing sVEGFR1 into the VEGF system was the predicted elevation of plasma VEGF, counter-intuitive to experimental suggestions that sVEGFR1 lowers the availability of circulating VEGF *in vivo* [8], [16], [18]. Specifically, tuning [sR1]*_pl_* up from 0 to 200 pM was accompanied by corresponding increases in [V]*_pl_* from 1 to 2 pM (Fig. 3). Analyzing the flow diagrams of the soluble species (Fig. 4), we postulated that sVEGFR1 – as a diffusible decoy receptor of VEGF – was able to facilitate transport of VEGF from the tissue interstitium into blood via either lymph or vascular permeability flow. It is thus conceivable that other soluble receptors of VEGF with interstitial origins could similarly facilitate interstitia-to-blood transport of VEGF – effectively lowering interstitial VEGF and elevating plasma VEGF – allowing us to target a [V]*_pl_* of 1.5 pM using lower VEGF-secretion rates and consequentially lower [V]*_IS_*. Possible candidates include: (a) human soluble VEGFR2 (160 kDa) – present in significant quantities in healthy human plasma (7–8 ng/mL) [140] and upregulated in acute myeloid leukemia [39]; (b) soluble NRP1 (90 kDa) – a VEGF_165_-specific antagonist, with documented renal expression in humans [141], [142]; and (c) cellular fibronectin (∼500 kDa) – with VEGF-affinity sites on its heparin-binding domain [124], [143] and normally present in extracellular matrix but can end up in elevated amounts in plasma upon endothelial dysfunction or vascular injury, such as in pre-eclampsia [144].

Furthermore, our model currently has no blood sources of VEGF or sVEGFR1, which would elevate their respective plasma concentrations. Thrombin-activated platelets have been known to release VEGF from their α-granules [145], as well as fibronectin-VEGF complexes [143], during wound-healing angiogenesis. Activated peripheral blood mononuclear cells may also be a direct blood source of sVEGFR1 [17]. Intramuscular production of sVEGFR1 was assumed in this model to occur entirely through abluminal endothelial secretion into the interstitium; luminal endothelial secretion into the circulation is a conceivable direct blood source of sVEGFR1 but has yet to be quantitatively documented.

Moreover, interstitial proteolytic clearance of soluble or matrix-bound VEGF – e.g., plasmin- and matrix metalloproteinase (MMP)-mediated cleavage of VEGF_165_ and the respective release of VEGF_110_ and VEGF_113_ fragments [95], [146], as well as the intrinsic protein degradation rate of VEGF [147] – was not considered in the current model. Release of VEGF_165_ from interstitial matrix sites through plasmin/MMP degradation of matrix core proteins was also neglected. Abnormal plasma levels of MMPs in PAD [148], [149] may suggest a role for proteolytic degradation or release of VEGF_165_ in causing pathological bioavailabilities of VEGF. The recent discovery of possible modulation by MMPs on sVEGFR1's effects on VEGF-VEGFR2 signaling was also not considered here [150].

Lastly, there may be specialized organs with higher VEGF production rates which, if separately partitioned from our normal compartment, may lessen the burden on skeletal muscle for VEGF production (i.e., VEGF release from tissue to blood, whether via lymph or vascular permeability). Other adult cell sources where VEGF expression has been qualitatively described [151] include: (i) epithelial cells near fenestrated blood vessels (high vascular permeability), e.g., choroid plexus and kidney glomeruli; and (ii) cells adjacent to sinusoidal vessels, e.g., in the liver (which produces half of all lymph formed by the body at rest [93]) and spleen (also the main site of destruction of platelets [93]). In other words, the high interstitial VEGF level predicted in our normal compartment may currently be skewed by lumping skeletal muscle with these organs of higher interstitial VEGF levels.

### Introduction of homodimerized sVEGFR1 and lymphatic drainage did not drastically alter VEGF ligand and receptor distributions in healthy muscle tissues

The model extensions to include homodimeric sVEGFR1 and lymphatic biotransport did not significantly alter our prior steady-state predictions for healthy muscle tissues [54], [59]: that most extracellular VEGF were surface receptor- or matrix-bound; that almost all matrix binding sites were unoccupied by VEGF or sVEGFR1; and that less than a quarter of total receptors on the abluminal surface (8% of VEGFR1, 20% of VEGFR2, and 18% of NRP1) were part of VEGF-bound signaling complexes. In its capacity as a soluble or NRP1-bound trap of VEGF, sVEGFR1 only held small fractions of total interstitial VEGF at control: 0.7% and 3.25% in the normal and calf compartments respectively. However, the present study did not rule out the possibility that monomeric sVEGFR1, in its capacity to heterodimerize with surface VEGFR1 or VEGFR2 monomers, can significantly alter these distributions.

### Significant complexed fractions of VEGF and sVEGFR1 in plasma necessitate re-evaluation of assay specificity

At our simulated healthy control, 77% of total plasma VEGF was sVEGFR1-complexed and 5% of total sVEGFR1 was VEGF-bound, both considerably higher than experimentally measured [45]. The much lower complexed fractions from experimental data may indicate that *in vivo*, other soluble receptors (e.g., sVEGFR2, sNRP1, plasma fibronectin) can strongly compete with sVEGFR1 as plasma reservoirs for VEGF, or significant quantities of other ligands (e.g., PlGF, VEGF-B) are present to compete with VEGF for sVEGFR1 binding. We propose that the experimental quantification of all major *in vivo* binding partners for both VEGF and sVEGFR1 from peripheral blood samples to be essential in reconciling the apparent contradiction between predicted and measured VEGF-sVEGFR1 complexed fractions. Alternatively, if our computational predictions correctly suggest that the complexed fractions of VEGF and sVEGFR1 were underestimated experimentally, the implications are that: (1) measurements of free VEGF and free sVEGFR1 should not be taken as stand-ins for circulating (free+bound) VEGF and sVEGFR1 levels [45], and may be insufficient in characterizing disease states; and (2) the variation in complexed fractions may partially account for the well-documented heterogeneity in measurements of plasma VEGF and sVEGFR1. Re-evaluations of assay specificity and protocol standardization for measuring free vs. complexed sVEGFR1, as was done for VEGF [152], may be worthwhile.

### Simulation of VEGF-trapping did not recapitulate sVEGFR1's anti-angiogenic potential

Several experimental studies have demonstrated the ability of sVEGFR1 as a ligand sink in lowering the availability of free VEGF *in vitro*, *ex vivo* and *in vivo* [8], [16], [153], or have implicated this ligand-trapping mechanism of sVEGFR1 in the pathogenesis of disease states where inverse changes between VEGF and sVEGFR1 concentrations were generally observed [18]. Thus it was surprising that in our simulations, upregulated expression of either VEGF or sVEGFR1 was not accompanied by significant inverse changes in the systemic levels of the other, as intuitively expected of a straight-forward ligand-trapping behavior. Instead, increasing VEGF production rate from the normal tissue compartment led to drastic increases in sVEGFR1 levels systemically (Fig. 2A). Conversly, when sVEGFR1 production rates were increased, interstitial VEGF only decreased minutely while plasma VEGF counter-intuitively increased drastically (Fig. 3A).

Based on analyses of the flow balances and distribution profiles at control, we hypothesized that several confounding mechanisms were obscuring the most direct effects of VEGF-trapping by sVEGFR1 (i.e., where upregulated expression of one lowers the availability of the other). In the first scenario where VEGF production was increased, we postulated that increasing VEGF-occupancies of NRP1-coupled complexes actually reduced NRP1-dependent internalization of sVEGFR1, resulting in the non-intuitive increase in systemic levels of free sVEGFR1. In the second scenario where sVEGFR1 production was increased, we postulated that the transendothelial gradients at control were permissive to the trapping of interstitial VEGF by sVEGFR1 and the shuttling of the formed complexes into the blood, whereupon the subsequent dissociation resulted in the non-intuitive increase in plasma free VEGF. In other words, we hypothesized that the net tendency for sVEGFR1-VEGF complex association in the tissue interstitium provided an extra conduit for VEGF to be transported into the tissue (via its complexed form, in addition to simple intravasation of free VEGF; see Fig. 4). Thus an immediate future direction is to perform a thorough mechanistic study to computationally validate these hypotheses, and to search for the conditions under which the VEGF-trapping ability of sVEGFR1 can account for reductions in free VEGF within the same compartment and/or systemically.

Under the current simulation settings, however, a particularly important result was that the intensities of signaling complex formation (directly proportional to interstitial free VEGF levels) were negligibly affected by sVEGFR1 production rates (Fig. 3B) –computational simulation of just the VEGF-trapping interactions of sVEGFR1 was insufficient to replicate the expected inhibitory function of sVEGFR1 on VEGF-signaling. Counter to prevailing wisdom, simulated increase in production of sVEGFR1 did not lead to significant reduction in intramuscular VEGF-VEGFR2 complex formation. This implication that VEGF-trapping alone may not account for sVEGFR1's anti-angiogenic potential needs to be experimentally validated; while computationally, the next step would be to investigate whether heterodimerization with surface VEGFRs is the key to sVEGFR1's purported contribution in impaired angiogenesis.

Also worth considering are the functional consequences of the counter-intuitive increase in plasma VEGF, at the expense of interstitial VEGF, in response to sVEGFR1 production. While the current model did not simulate luminal endothelial surface VEGFRs, their potential *in vivo* presence (discussed further below) may suggest the unexpected angiogenic activation of microvasculature in distal tissues by the elevated plasma VEGF as a result of local intramuscular production of sVEGFR1.

### Changing levels of circulating sVEGFR1 may reflect altered tissue expression of surface receptors

In examining system responses to variations in receptor expression levels and effective ligand-receptor affinities from control values, we confirmed that the introduction of sVEGFR1 into the model did not change previous predictions [54] regarding NRP1 density as a functional “angiogenic switch” of VEGF signaling in skeletal muscle tissue (Fig. 3B). We also demonstrated that the newly proposed VEGF_121_-NRP1 affinity has a negligible effect on the VEGF signaling profiles (Fig. 4D,E). We further showed that NRP1-dependent internalization of sVEGFR1 can be a major regulator of free sVEGFR1 levels in both interstitia and plasma, both directly (e.g., varying NRP1 densities) and indirectly (e.g., R2/R1 ratio and VEGF-VEGFR1 binding alter the availability of uncoupled NRP1s) (Fig. 5A, 6B). The clinical implication is that pathological levels of plasma sVEGFR1 may not necessarily indicate altered sVEGFR1 production or altered availability of free VEGF, because they may partially reflect altered receptor expression in tissues. This will likely be even more significant when sVEGFR1-heterodimerization with surface receptors are considered.

### Implications of model assumptions for surface receptors

Here we discuss several assumptions made in the current modeling of surface receptors – (1) static receptor internalization and insertion (conservation of total receptor density), (2) no intracellular trafficking & signaling, (3) no receptor heterodimers, (4) no luminal surface receptors – and in particular, their relevance to the study of sVEGFR1.

Firstly, our current implementation assumed constant internalization rates for all receptors and complexes, paired with constant free receptor insertion rates (free VEGFR1, free VEGFR2, free NRP1) defined to conserve the total receptor densities (total VEGFR1, total VEGFR2, total NRP1) at the cell surface. This conservation assumption – i.e., the artificial maintenance of a fixed total receptor density irrespective of the interstitial free VEGF concentration or the degree of receptor activation – had implicitly driven the magnitudes of the internalization flows of soluble species in the tissue compartments. Future investigation is needed to quantify whether the large values of VEGF internalization flows predicted in this study critically depended on the modeled internalization rates and conservation assumptions. Adjustments to the internalization flows of VEGF could significantly alter the secretion flows of VEGF needed to maintain the same steady-state interstitial VEGF concentrations, and in turn significantly shift the tissue flow balance on Fig. 4, such that interstitial VEGF might acquire acute sensitivity to transport parameters, as well as to depletory ligand-trapping by sVEGFR1, against our current findings. There is also justification for beginning investigations into dynamic regulation of receptor internalization, in particular for VEGFR2: VEGF-induction of VEGFR2 internalization has been shown in various studies as receptor tyrosine kinase (RTK) autophosphorylation-, dynamin-, clathrin- and VEGFR1-mediated processes [154]–[157]. Particularly relevant is the latter process of regulation through VEGFR1-VEGFR2 crosstalk, which possibly presents an opportunity for sVEGFR1, as a VEGFR1 competitor, to moderate VEGFR2 activation and internalization.

Secondly, our network of biochemical interactions ended at the level of ligand-receptor binding and complex internalization. Thus far we have used the cell surface densities of VEGF-VEGFR complexes as surrogate markers for “angiogenic signaling potential” [158]; however, there is increasing experimental information on intracellular VEGF signaling, whose contribution can only be assessed with detailed model extensions to keep track of endosomal and caveolar populations of internalized complexes, as well as VEGF-regulated sorting towards degradation, nuclear translocation, or recycling back to the cell surface [159], [160].

Thirdly, there is recent *in vitro* and *in silico* evidence supporting significant prevalence of VEGFR1-VEGFR2 heterodimers on endothelial cell surfaces, with signaling properties distinct from those of VEGFR homodimers [68], [70], [94]. Receptor heterodimers were neglected in this model for the lack of *in vivo* quantification of their relative abundance, and would not have affected current predictions of sVEGFR1's effects on the VEGF system until the focus of study switches to sVEGFR1's alternate role in heterodimerizing with surface VEGFRs.

Lastly, in this study, all receptors were confined to the abluminal endothelial cell surface, in congruence to experimental studies inferring abluminal localization of VEGFRs based on VEGF immunostaining [161] and the common assumption that VEGFRs signaling respond more to local/interstitial VEGF concentrations rather than circulating/plasma VEGF [152]. However, this assumption needs to be thoroughly re-evaluated, in light of recent VEGFR2-immunostaining that localized VEGFR2 equally on luminal and abluminal surfaces of tumor- and adenoVEGF-induced microvascular endothelium, as well as significantly on transendothelial vesiculovaculolar organelles (VVOs) and luminally-attached caveolae [162]. It is unclear whether VEGFR1 and NRP1 also have significant luminal populations, or whether luminal redistribution follows the caveolin-1-dependent internalization unique to VEGFR2 [158]. If luminal expression of VEGFR1 is considered in the future, luminal secretion of sVEGFR1 should be simultaneously investigated as well. Interestingly, a possible luminal bias in Nedd4-stimulated VEGFR2 degradation has also been postulated [158]. Although it is uncertain whether luminal vs. abluminal VEGFR2 have equal signaling potential or similar downstream targets [162], there is urgent need to explore the possibility, especially in the next step of investigating sVEGFR1-heterodimerization with surface receptors, i.e., given the much higher predicted sVEGFR1 concentration in plasma than in the interstitium, the luminal extent of sVEGFR1-VEGFR heterodimerization would be expected to exceed that on the abluminal side.

### sVEGFR1 may attenuate interstitial matrix-bound VEGF gradients by 10% at most

We showed that the densities and affinities of the interstitial matrix binding sites did not affect steady-state predictions of plasma and interstitial levels of the soluble species, nor VEGFR occupancies. We also showed consistently minuscule fractional occupancies of total matrix proteoglycan sites, i.e., VEGF and sVEGFR1 proteins are always hugely outnumbered by glycosaminoglycan binding-sites in the interstitial matrix. To the extent that the present study focuses on sVEGFR1's effect on the formation of surface VEGF signaling complexes, the uncertainties in the characterization of matrix sites are not of concern. However, the absolute quantities of matrix-bound VEGF_165_ and sVEGFR1 – comprising 36% and 72% of total VEGF_165_ in the normal and calf tissues respectively, as well as 97% of total sVEGFR1 in either tissue – were greatly sensitive to matrix site densities and affinities (Fig. 7). The sizes of matrix-bound reservoirs of VEGF and sVEGFR1 may affect the time course of dynamic concentration changes in free VEGF, free sVEGFR1 or surface signaling complexes. Although the compartmental model did not include the spatial resolution needed to examine interstitial VEGF gradients, the *in vivo* distribution of matrix sites between the ECM and BMs could affect spatial morphogenic gradients that guide sprouting angiogenesis. In fact, a recent study attributed the ability of sVEGFR1 in rescuing the branching morphogenesis of developing vessels in VEGFR1^−/−^ mutant embryonic cell cultures to sVEGFR1's role as a diffusible VEGF sink: that by diffusing away from the cell surface, it is able to alter the interstitial VEGF gradients as presented to endothelial tip cell filopodia of sprouting vessels [163].

The present model was not able to directly address the modulating effects of sVEGFR1 on the matrix-bound VEGF gradients, because we did not model the subsequent binding of sVEGFR1 to matrix-bound VEGF (forming *M·V·sR_1_*), due to insufficient experimental knowledge about its hypothetical binding configuration – e.g., whether the sVEGFR1-binding domain of VEGF is still exposed after matrix-association, and whether subsequent sVEGFR1 binding can effectively mask or protect the matrix-bound VEGF from participating in capillary sprout guidance. However, considering that the ratio of interstitial free sVEGFR1 to matrix-bound VEGF was ∼1∶12.5 (Fig. S2), even the hypothetical recruitment of all available sVEGFR1 would only attenuate the matrix gradient of VEGF by ∼10% (non-equilibrated). Ignoring all other compensating interactions and processes in the molecular system, and assuming *K_d_*(*M·V*,*sR_1_*) ∼*K_d_*(*V*,*sR_1_*), a simple equilibrium approximation of sVEGFR1 attenuation of matrix-bound VEGF would be ∼7.5%:
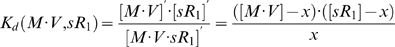


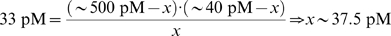


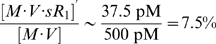



### Physiological variations in transport rates affected the stability and reliability of plasma VEGF and sVEGFR1 as diagnostic markers

We showed that both free VEGF and free sVEGFR1 levels in plasma varied significantly over the tested physiological ranges of transport parameters: e.g., the activity-dependent *k_L_* changes typically experienced during an active day led to fluctuations in [sR1]*_pl_* and [V]*_pl_* of up to 76 pM and 1.65 pM about their healthy control levels of 100 pM and 1.5 pM respectively (Fig. 9B). This indicated that normal inter-patient variations in permeability and lymph flow rates – as dependent on patients' physical activity levels (sedentary vs. active lifestyle), posture during blood sampling, etc. – may contribute to the physiological heterogeneity in the clinical measurements of plasma VEGF and sVEGFR1 among healthy control cohorts. Corrections for these factors may yield more stable and consistent healthy benchmarks for the use of plasma VEGF or sVEGFR1 as diagnostic markers.

On the other hand, interstitial VEGF levels remained unchanged over the physiological variations in transport rates, because the predicted secretion and internalization flows of VEGF dominated over all its inter-compartmental transport flows. These relative flow magnitudes have not been experimentally validated; *in vivo* quantification of VEGF secretion rates would be invaluable to assess our targeted secretion rates which were highly dependent on the chosen initial configurations of internalizable receptors. When interstitial VEGF is unresponsive to changes in transport rates, so is the predicted formation of signaling complexes on the tissue endothelia. The significance here is that the physiological variation in transport rates offers a case example where the common circulating angiogenic markers become unreliable, i.e., apparent changes in free VEGF or sVEGFR1 levels in the plasma do not correlate with changes in the actual angiogenic signaling potential in the tissues. However, considering the significant sensitivity of interstitial sVEGFR1 to transport rates, we cannot rule out the possibility that once sVEGFR1-heterodimerization with surface VEGFRs is in place, that transport rates may indeed affect the formation of signaling complexes in muscles.

As a first assessment of whether activity-dependent *k_L_* can account for the surge in plasma sVEGFR1 observed after acute exercise in healthy individuals, simulations were performed without concurrent exercise-induced changes to sVEGFR1 or VEGF production, *k_P_*, or receptor expression. Immediately after the onset of exercise, independent simulation of activity-dependent *k_L_* predicted that an elevation in [V]*_pl_* preceded the larger but slower elevation in [sR1]*_pl_* (Fig. 9B). In contrast, a human study [49] showed acute exercise to cause a fast ∼50% elevation in [sR1]*_pl_* (peaking ∼0.5 h after exercise) which preceded a temporary ∼50% drop in [V]*_pl_* (lowest at ∼2 h after exercise). Thus our simulated *k_L_*-induced increase in [sR1]*_pl_* roughly matched that in the exercise study in magnitude but not in speed; while our *k_L_*-induced changes in [V]*_pl_* was in opposite direction to those in the exercise study. This may suggest that hypoxia-induced secretion of sVEGFR1 (as opposed to lymphatic drainage of sVEFR1) is indeed the faster and major source of elevated plasma sVEGFR1 in exercise; but our simulations were unable to confirm the conclusion by Bailey *et al.* [49] that VEGF trapping by the surge of sVEGFR1 had caused the drop in plasma VEGF. In any case, the fact that lower *k_L_*'s associated with inactivity led to lower plasma sVEGFR1 (Fig. 9B) supported the possibilities that: (i) PAD-associated immobility may contribute to the lower plasma sVEGFR1 seen in PAD patients, although it remains to be experimentally validated whether a corresponding accumulation of interstitial sVEGFR1 and consequential impairment of muscle angiogenesis would occur; and that (ii) exercise rehabilitation may partly reverse the pathological levels of circulating angiogenic markers through activity-induced *k_L_*, thereby ameliorating the angiogenic state in PAD patients. If future considerations of sVEGFR1 heterodimerization with surface VEGFR can establish a link between transport-dependent fluctuations in sVEGFR1 levels and angiogenic signalling intensity, the clinical implications would be significant: e.g., therapeutic delivery of VEGF-C/D (the VEGF family members involved in lymphangiogenesis) alongside VEGF-A to ischemic tissues could be explored for synergistic effects of increased lymph flow on the angiogenic efficacy of VEGF-A.

Limitations in model assumptions that can affect the above conclusions include: underestimations of the effective control *k_L_* and *k_P_* due to lumping of highly-perfused organs with skeletal muscle tissue in the normal compartment; overestimation of whole-body exercise-induced increases in *k_L_* and *k_P_* as non-skeletal muscle organs (e.g., liver) should maintain resting rates; and misestimation of clearance rates of sVEGFR1 and sVEGFR1-VEGF from the plasma half-lives of synthetic soluble VEGF-traps.

### Future directions

The immediate task is to perform a computational mechanistic study to investigate why simulations of VEGF-trapping by sVEGFR1 did not demonstrate any purported anti-angiogenic effects: Does interstitium-to-blood shuttling of VEGF-sVEGFR1 complexes explain the apparent absence of local reductions in free VEGF upon increased interstitial sVEGFR1 production? Are there alternate conditions under which sVEGFR1, as a VEGF sink, can indeed dampen VEGF signaling as *in vitro* and *ex vivo* experiments have suggested?

sVEGFR1-heterodimerization of surface VEGFRs is expected to present an alternative way for sVEGFR1 to modulate VEGF signal transduction even when interstitial free VEGF levels remain high, by effectively reducing the availability of functional endothelial surface VEGFR dimers for VEGF activation, assuming that VEGFR heterodimers with sVEGFR1 cannot signal. These effects are likely to be heavily determined by the proportionality of sVEGFR-VEGFR1 vs. sVEGFR-VEGFR2 binding; that is, any bias in the coupling of sVEGFR1 to VEGFR1 or VEGFR2 would tilt VEGF-activated signaling in the direction of the other receptor. Thus the next step would be to extend the model to quantify the contribution of this heterodimerization mechanism to sVEGFR1's anti-angiogenic potential.

Table 12 compares our significant computational predictions with current experimental data; future experiments to address the proposed sources of discrepancies are recommended. With future availability of experimental validation to address the current limitations in model assumptions, the multi-compartmental model put forth in this study can be more finely tuned to accurately represent human subjects – healthy or diseased – to serve as a computational platform for design and testing of integrative pro-angiogenic therapies.

**Table 12:** Comparing Computational Predictions with Experimental Data.

Computational Predictions	Experimental Data	Possible Explanations of Discrepancy
Interstitial VEGF in muscle, [V]*_IS_* = 10 pM	[V]*_IS_*∼1 pM based on microdialysis [135], [136]	Technical issues with macromolecular measurements using microdialysis; Model overestimation of sVEGFR1-facilitated transport of VEGF; Missing blood sources of VEGF in model.
77% of total plasma VEGF was sVEGFR1-complexed	∼4% mole fraction [45]	Other unmodeled soluble receptors (e.g., sVEGFR2, sNRP1, plasma fibronectin) compete for VEGF *in vivo*.
5% of total plasma sVEGFR1 was VEGF-bound	∼0.65% mole fraction [45]	Other unmodeled ligands (e.g., PlGF, VEGF-B) compete for sVEGFR1 *in vivo*.
sVEGFR1 as a ligand sink negligibly reduced interstitial free VEGF while drastically elevating plasma free VEGF	sVEGFR1 as a ligand sink lowers availability of free VEGF *in vitro*, *ex vivo* and *in vivo* [8], [16], [153]	Computational model examined transport between tissue and blood compartments; Experimental setups examined single-compartment (e.g. pooled amniotic fluids) or relatively closed system (e.g., avascular cornea) systems.
sVEGFR1 did not reduce intramuscular VEGF-VEGFR2 complex formation	sVEGFR1 is anti-angiogenic (cornea [16], pre-eclampsia [17], [18], cancer [21]–[30])	Current computational model neglected sVEGFR1-heterodimerization with surface VEGFRs.
Exercise-induced lymph flow rates elevated plasma VEGF faster than plasma sVEGFR1	Acute exercise quickly elevated plasma sVEGFR1, then reduced plasma VEGF [49]	Other exercise-induced parameter changes (e.g., hypoxia-induced sVEGFR1 production) not modeled computationally.

## Supporting Information

Figure S1 Healthy Subject: Complete Molecular Distribution Analysis for Varying VEGF Secretion Rates. (A) Free VEGF and free sVEGFR1 distributions. (B) Total sVEGFR1 distribution. (C) Total VEGF121 distribution. (D) Total VEGF165 distribution. (E) Extracellular matrix binding site occupancies. (F) Endothelial basement membrane binding site occupancies. (G) Parenchymal basement membrane binding site occupancies. (H) VEGFR1 occupancies. (I) VEGFR2 occupancies. (J) NRP1 occupancies. (K) VEGF-bound VEGFR complexes.
(0.15 MB PDF)

Figure S2 Healthy Subject: Complete Molecular Distribution Analysis for Varying sVEGFR1 Secretion Rates. (A) Free VEGF and free sVEGFR1 distributions. (B) Total sVEGFR1 distribution. (C) Total VEGF121 distribution. (D) Total VEGF165 distribution. (E) Extracellular matrix binding site occupancies. (F) Endothelial basement membrane binding site occupancies. (G) Parenchymal basement membrane binding site occupancies. (H) VEGFR1 occupancies. (I) VEGFR2 occupancies. (J) NRP1 occupancies. (K) VEGF-bound VEGFR complexes.
(0.15 MB PDF)
