# Supplementary material for: A Compartment Model of VEGF Distribution in Humans in the Presence of Soluble VEGF Receptor-1 Acting as a Ligand Trap
Source: PLoS One. 2009 Apr 8;4(4):e5108. doi: 10.1371/journal.pone.0005108 (PMC2663039; doi:10.1371/journal.pone.0005108)
Supplement: Figure S2 — Healthy Subject: Complete Molecular Distribution Analysis for Varying sVEGFR1 Secretion Rates. (A) Free VEGF and free sVEGFR1 distributions. (B) Total sVEGFR1 distribution. (C) Total VEGF121 distribution. (D) Total VEGF165 distribution. (E) Extracellular matrix binding site occupancies. (F) Endothelial basement membrane binding site occupancies. (G) Parenchymal basement membrane binding site occupancies. (H) VEGFR1 occupancies. (I) VEGFR2 occupancies. (J) NRP1 occupancies. (K) VEGF-bound VEGFR complexes. (0.15 MB PDF) [file pone.0005108.s002.pdf]

Healthy Subject: Varying sVEGFR1 secretion rate, q_{sR1} (molecule/EC/s)

q_{sR1}	Normal	Calf
	[molecule/EC/sec ; mole/(cm³ tissue)/sec]	
**Max**	2.15×10^{-2} ; 2.61×10^{-19}	4.30×10^{-2} ; 3.14×10^{-19}
**High**	1.60×10^{-2} ; 1.94×10^{-19}	3.20×10^{-2} ; 2.34×10^{-19}
**Healthy Ctrl**	1.07×10^{-2} ; 1.30×10^{-19}	2.10×10^{-2} ; 1.53×10^{-19}
**Low**	5.50×10^{-3} ; 6.67×10^{-20}	1.10×10^{-2} ; 8.04×10^{-20}
**Min**	6.00×10^{-5} ; 7.27×10^{-22}	1.20×10^{-4} ; 8.77×10^{-22}
**No sR1**	0	0

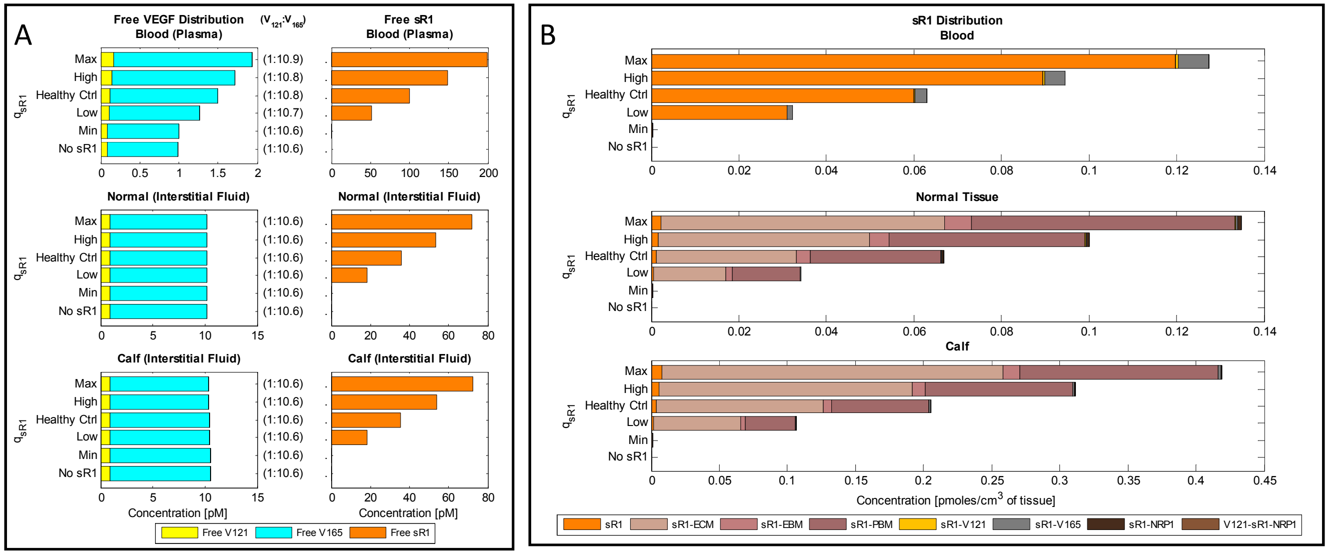

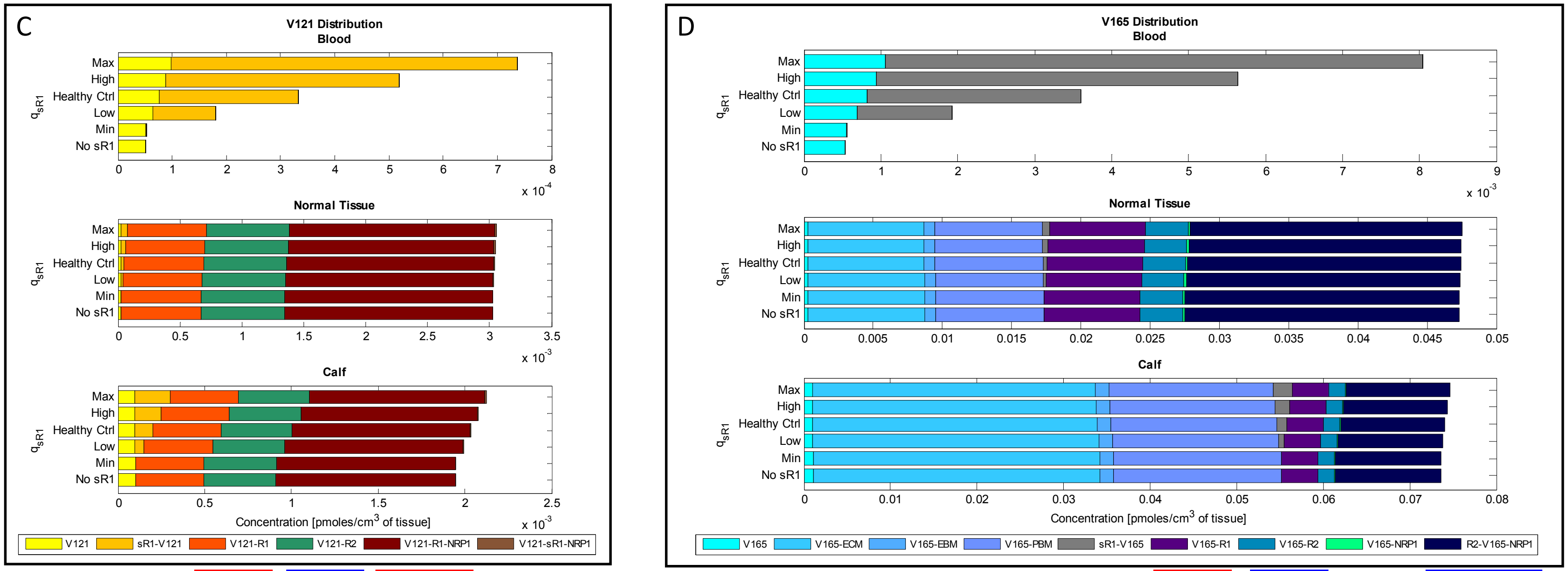

Supplemental Figure S2. Healthy Subject: Complete Molecular Distribution Analysis for Varying sVEGFR1 Secretion Rates. (A) Free VEGF and free sVEGFR1 distributions. **(B)** Total sVEGFR1 distribution. **(C)** Total $VEGF_{121}$ distribution. **(D)** Total $VEGF_{165}$ distribution. **(continued on next page)**

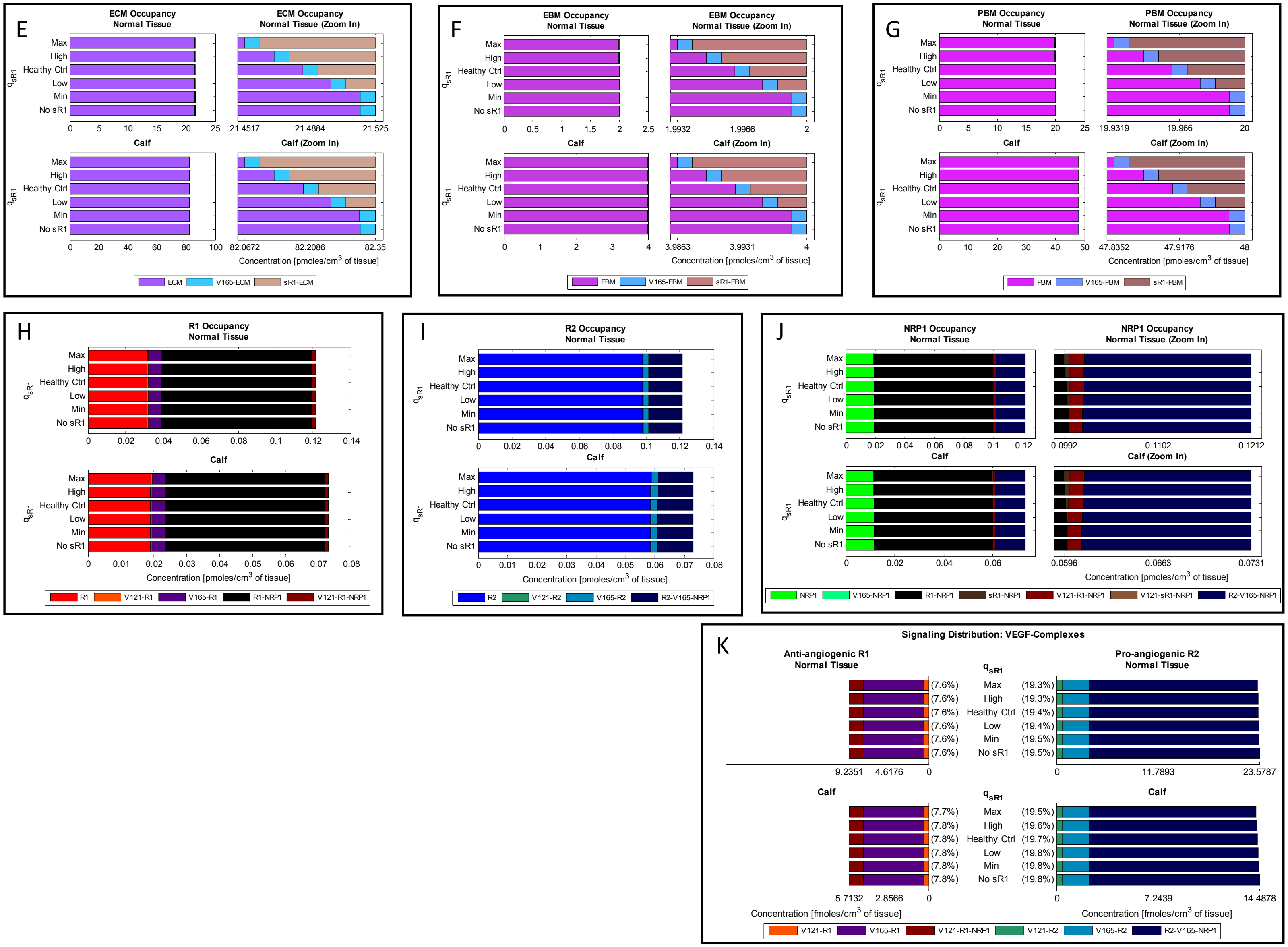

Supplemental Figure S2. (continued)

(E) Extracellular matrix binding site occupancies. **(F)** Endothelial basement membrane binding site occupancies. **(G)** Parenchymal basement membrane binding site occupancies. **(H)** VEGFR1 occupancies. **(I)** VEGFR2 occupancies. **(J)** NRP1 occupancies. **(K)** VEGF-bound VEGFR complexes.
